# AI recognition of patient race in medical imaging: a modelling study

**DOI:** 10.1016/S2589-7500(22)00063-2

**Published:** 2022-05-11

**Authors:** Judy Wawira Gichoya, Imon Banerjee, Ananth Reddy Bhimireddy, John L Burns, Leo Anthony Celi, Li-Ching Chen, Ramon Correa, Natalie Dullerud, Marzyeh Ghassemi, Shih-Cheng Huang, Po-Chih Kuo, Matthew P Lungren, Lyle J Palmer, Brandon J Price, Saptarshi Purkayastha, Ayis T Pyrros, Lauren Oakden-Rayner, Chima Okechukwu, Laleh Seyyed-Kalantari, Hari Trivedi, Ryan Wang, Zachary Zaiman, Haoran Zhang

**Affiliations:** **Department of Radiology** (J W Gichoya MD, A R Bhimireddy MS, H Trivedi MD) **and Department of Computer Science** (Z Zaiman), **Emory University, Atlanta, GA, USA; School of Computing, Informatics, and Decision Systems Engineering, Arizona State University, Tempe, AZ, USA** (I Banerjee PhD, R Correa BS); **School of Informatics and Computing, Indiana University–Purdue University, Indianapolis, IN, USA** (J L Burns MS, S Purkayastha PhD); **Institute for Medical Engineering and Science** (L A Celi MD, M Ghassemi PhD) **and Department of Electrical Engineering and Computer Science** (M Ghassemi), **Massachusetts Institute of Technology, Cambridge, MA, USA; Department of Medicine, Beth Israel Deaconess Medical Center, Boston, MA, USA** (L A Celi); **Department of Computer Science, National Tsing Hua University, Hsinchu, Taiwan** (L-C Chen BS, P-C Kuo PhD, R Wang BS); **Department of Computer Science, University of Toronto, Toronto, ON, Canada** (N Dullerud MS, L Seyyed-Kalantari PhD, H Zhang MS); **Stanford University School of Medicine, Palo Alto, CA, USA** (S-C Huang, M P Lungren MD); **Australian Institute for Machine Learning** (L Oakden-Rayner MD, L J Palmer PhD) **and School of Public Health** (L J Palmer), **University of Adelaide, Adelaide, SA, Australia; Florida State University College of Medicine, Tallahassee, FL, USA** (B J Price MD); **Dupage Medical Group, Hinsdale, IL, USA** (A T Pyrros MD); **Department of Computer Science, Georgia Institute of Technology, Atlanta, GA, USA** (C Okechukwu MS); **Lunenfeld-Tanenbaum Research Institute, Sinai Health, Toronto, ON, Canada** (L Seyyed-Kalantari); **Vector Institute for Artificial Intelligence, Toronto, ON, Canada** (L Seyyed-Kalantari)

## Abstract

**Background:**

Previous studies in medical imaging have shown disparate abilities of artificial intelligence (AI) to detect a person’s race, yet there is no known correlation for race on medical imaging that would be obvious to human experts when interpreting the images. We aimed to conduct a comprehensive evaluation of the ability of AI to recognise a patient’s racial identity from medical images.

**Methods:**

Using private (Emory CXR, Emory Chest CT, Emory Cervical Spine, and Emory Mammogram) and public (MIMIC-CXR, CheXpert, National Lung Cancer Screening Trial, RSNA Pulmonary Embolism CT, and Digital Hand Atlas) datasets, we evaluated, first, performance quantification of deep learning models in detecting race from medical images, including the ability of these models to generalise to external environments and across multiple imaging modalities. Second, we assessed possible confounding of anatomic and phenotypic population features by assessing the ability of these hypothesised confounders to detect race in isolation using regression models, and by re-evaluating the deep learning models by testing them on datasets stratified by these hypothesised confounding variables. Last, by exploring the effect of image corruptions on model performance, we investigated the underlying mechanism by which AI models can recognise race.

**Findings:**

In our study, we show that standard AI deep learning models can be trained to predict race from medical images with high performance across multiple imaging modalities, which was sustained under external validation conditions (x-ray imaging [area under the receiver operating characteristics curve (AUC) range 0·91–0·99], CT chest imaging [0·87–0·96], and mammography [0·81]). We also showed that this detection is not due to proxies or imaging-related surrogate covariates for race (eg, performance of possible confounders: body-mass index [AUC 0·55], disease distribution [0·61], and breast density [0·61]). Finally, we provide evidence to show that the ability of AI deep learning models persisted over all anatomical regions and frequency spectrums of the images, suggesting the efforts to control this behaviour when it is undesirable will be challenging and demand further study.

**Interpretation:**

The results from our study emphasise that the ability of AI deep learning models to predict self-reported race is itself not the issue of importance. However, our finding that AI can accurately predict self-reported race, even from corrupted, cropped, and noised medical images, often when clinical experts cannot, creates an enormous risk for all model deployments in medical imaging.

**Funding:**

National Institute of Biomedical Imaging and Bioengineering, MIDRC grant of National Institutes of Health, US National Science Foundation, National Library of Medicine of the National Institutes of Health, and Taiwan Ministry of Science and Technology.

## Introduction

Bias and discrimination in artificial intelligence (AI) systems has been studied in multiple domains,^1–4^ including in many health-care applications, such as detection of melanoma,^5,6^ mortality prediction,^7^ and algorithms that aid the prediction of health-care use,^8^ in which the performance of AI is stratified by self-reported race on a variety of clinical tasks.^9^ Several studies have shown disparities in the performance of medical AI systems across race. For example, Seyyed-Kalantari and colleagues showed that AI models produce significant differences in the accuracy of automated chest x-ray diagnosis across racial and other demographic groups, even when the models only had access to the chest x-ray itself.^9^ Importantly, if used, such models would lead to more patients who are Black and female being incorrectly identified as healthy compared with patients who are White and male. Moreover, racial disparities are not simply due to under-representation of these patient groups in the training data, and there exists no statistically significant correlation between group membership and racial disparities.^10^

In related work, several groups reported that AI algorithms can identify various demographic patient factors. One study^11^ found that an AI model could predict sex and distinguish between adult and paediatric patients from chest x-rays, while other studies^12^ reported reasonable accuracy at predicting the chronological age of patients from various imaging studies. In ophthalmology, retinal images have been used to predict sex, age, and cardiac markers (eg, hypertension and smoking status).^13–15^ These findings, which show that demographic factors that are strongly associated with disease outcomes (eg, age, sex, and racial identity), are also strongly associated with features of medical images and might induce bias in model results, mirroring what is known from over a century of clinical and epidemiological research on the importance of covariates and potential confounding.^16,17^ Many published AI models have conceptually amounted to simple bivariate analyses (ie, image features and their ability to predict clinical outcomes). Although more recent AI models have begun to consider other risk factors that conceptually approach multivariate modelling, which is the mainstay of clinical and epidemiological research, key demographic covariates (eg, age, sex, and racial identity) have been largely ignored by most deep learning research in medicine.

Findings regarding the possibility of confounding of racial identity in deep learning models suggest a possible mechanism for racial disparities resulting from AI models: that AI models can directly recognise the race of a patient from medical images. However, this hypothesis is largely unexplored^18^ and, in contrast to other demographic factors (eg, age and sex), there is a widely held, but tacit, belief among radiologists that the identification of a patient’s race from medical images is almost impossible, and that most medical imaging tasks are essentially race agnostic (ie, the task is not affected by the patient’s race). Given the possibility for discriminatory harm in a key component of the medical system that is assumed to be race agnostic, understanding how race has a role in medical imaging models is of high importance^19^ as many AI systems that use medical images as the primary inputs are being cleared by the US Food and Drug Administration and other regulatory agencies.^20–22^

In this study, we aimed to investigate how AI systems are able to detect a patient’s race to differing degrees of accuracy across self-reported racial groups in medical imaging. To do so, we aimed to investigate large publicly and privately available medical imaging datasets to examine whether AI models are able to predict an individual’s race across multiple imaging modalities, various datasets, and diverse clinical tasks.

## Methods

### Definitions of race and racial identity

Race and racial identity can be difficult attributes to quantify and study in health-care research^23^ and are often incorrectly conflated with biological concepts (eg, genetic ancestry).^24^ In this modelling study, we defined race as a social, political, and legal construct that relates to the interaction between external perceptions (ie, “how do others see me?”) and self-identification, and specifically make use of self-reported race of patients in all of our experiments. We variously use the terms race and racial identity to refer to this construct throughout this study.

### Datasets

We obtained public and private datasets (table 1, appendix p 2) that covered several imaging modalities and clinical scenarios. No one single race was consistently dominant across the datasets (eg, the proportion of Black patients was between 6% and 72% across the datasets). For all datasets, ethical approval was obtained from the relevant institutional ethical boards.

### Investigation of possible mechanisms of race detection

We conduced three main groups of experiments to investigate the cause of previously established AI performance disparities by patient race. These experiments were: (1) to assess the ability of deep learning AI models to recognise race from medical images, including the ability of these models to generalise to new environments and across multiple imaging modalities; (2) to examine possible confounding anatomic and phenotype population features as explanations for these performance scores, and (3) to investigate the underlying mechanisms by which AI models can recognise race. The full list of experiments are summarised in table 2 and the appendix (pp 22–23).

We did not present measures of performance variance or null hypothesis tests because these data are uninformative given the large dataset sizes and the large effect sizes reported (ie, even in experiments in which a hypothesis could be defined, all p values were <0·001).

### Race detection in radiology imaging

To investigate the ability of deep learning systems to detect race from radiology images, first, we developed models for the detection of racial identity on three large chest x-ray datasets—MIMIC-CXR (MXR),^25^ CheXpert (CXP),^26^ and Emory-chest x-ray (EMX) with both internal validation (ie, testing the model on an unseen subset of the dataset used to train the model) and external validation (ie, testing the model on a completely different dataset than the one used to train the model) to establish baseline performance. Second, we trained racial identity detection models for non-chest x-ray images from multiple body locations, including digital radiography, mammograms, lateral cervical spine radiographs, and chest CTs, to evaluate whether the model’s performance was limited to chest x-rays.

After establishing that deep learning models could detect a patient’s race in medical imaging data, we generated a series of competing hypotheses to explain how this process might occur. First, we assessed differences in physical characteristics between patients of different racial groups (eg, body habitus^27^ or breast density^28^). Second, we assessed whether there was a difference in disease distribution among patients of different racial groups (eg, previous studies provide evidence that Black patients have a higher incidence of particular diseases, such as cardiac disease, than White patients).^29,30^ Third, we assessed whether there were location-specific or tissue-specific differences (eg, there is evidence that Black patients have a higher adjusted bone mineral density and a slower age-adjusted annual rate of decline in bone mineral density than White patients).^31,32^ Fourth, we assessed whether there were effects of societal bias and environmental stress on race outcomes from medical imaging data, as shown by differences in race detection by age and sex (reflecting cumulative and occupational differences in exposures). Last, we assessed whether there was an effect on the ability of AI deep learning systems to detect race when multiple demographic and patient factors were combined, including age, sex, disease, and body habitus.

We also investigated potential explanations of race detection that could target the known shortcut mechanisms that deep models might be using as proxies for race^33^ by evaluating, first, frequency domain differences in the high frequency image features (ie, textural) and low frequency image features (ie, structural) that could be predictive of race; second, how differences in image quality might influence the recognition of race in medical images (given the possibility that image acquisition practices might differ for patients with different racial identities); and, last, whether specific image regions contribute to the recognition of racial identity (eg, specific patches or regional variations in the images, such as radiographic markers in the top right corner).

### Role of the funding source

Grant support was used to pay for data collection, data analysis, data interpretation, and writing of the manuscript. The funders did not influence the decision to publish or the target journal for publication.

## Results

The deep learning models assessed in this study showed a high ability to detect patient race using chest x-ray scans, with sustained performance on other modalities and strong external validations across datasets (table 3).

The ability of deep learning models that were trained on the CXP dataset to predict patient race from the body-mass index (BMI) alone was much lower than the image-based chest x-ray models (area under the receiver operating characteristics curve [AUC] 0·55), indicating that race detection is not due to obvious anatomic and phenotypic confounder variables. Similar results were observed across stratified BMI groups (0·92–0·99; appendix p 24).

The ability of logistic regression models to classify race on the basis of tissue density (AUC 0·54) and on the combination of age and tissue density (0·61) was far lower than the ability of the image models on the breast mammograms in the EM-Mammo dataset (0·81; appendix p 25). These findings suggest that breast density and age did not account for most image model performance when detecting race.

Moreover, the ability of models to predict race from the diagnostic labels alone was much lower than the chest x-ray image-based models, with AUC values between 0·54 and 0·61 for MXR, and between 0·52 and 0·57 for CXP (appendix p 30). AUC values for race detection in the no finding class of 0·914 (95% CI 0·901–0·926) were obtained for Asian patients, 0·949 (0·945–0·953) for Black patients, and 0·941 (0·937–0·945) for White patients, versus 0·944 (0·938–0·950 [Asian patients]), 0·940 (0·937–0·942 [Black patients]), and 0·933 (0·930–0·936 [White patients]) for the entire dataset containing all disease classes, including the no finding class. These results suggest that high AUC values for racial identity recognition were not caused by disease labels.

We found that deep learning models effectively predicted patient race even when the bone density information was removed for both MXR (AUC value for Black patients: 0·960 [CI 0·958–0·963]) and CXP (AUC value for Black patients: 0·945 [CI 0·94–0·949]) datasets. The average pixel thresholds for different tissues did not produce any usable signal to detect race (AUC 0·5). These findings suggest that race information was not localised within the brightest pixels within the image (eg, in the bone).

For patients in different age groups, there was no appreciable difference in racial identity recognition performance (appendix p 15). Similarly, there was also no appreciable difference in racial identity recognition performance between male and female patients (appendix p 17).

The performance of a logistic regression model (AUC 0·65), a random forest classifier (0·64), and an XGBoost model (0·64) to classify race on the basis of age, sex, gender, disease, and body habitus performed much worse than the race classifiers trained on imaging data (AUC >0·95; appendix p 20). This finding suggests that the combination of these confounders did not significantly affect the imaging model’s ability to classify race.

**Figure 1:**
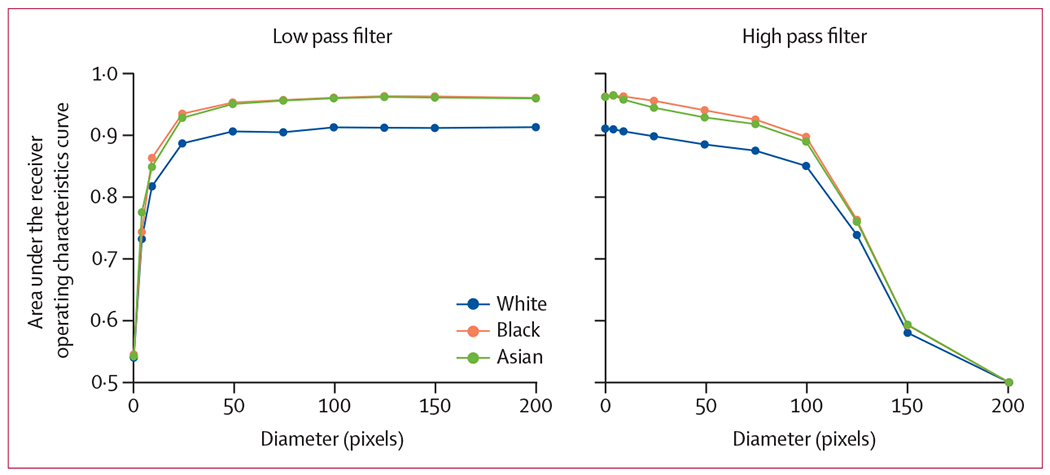
The effect on model performance of adding a low-pass filter and a high-pass filter for various diameters in the MXR dataset MXR=MIMIC-CXR dataset.

**Figure 2:**
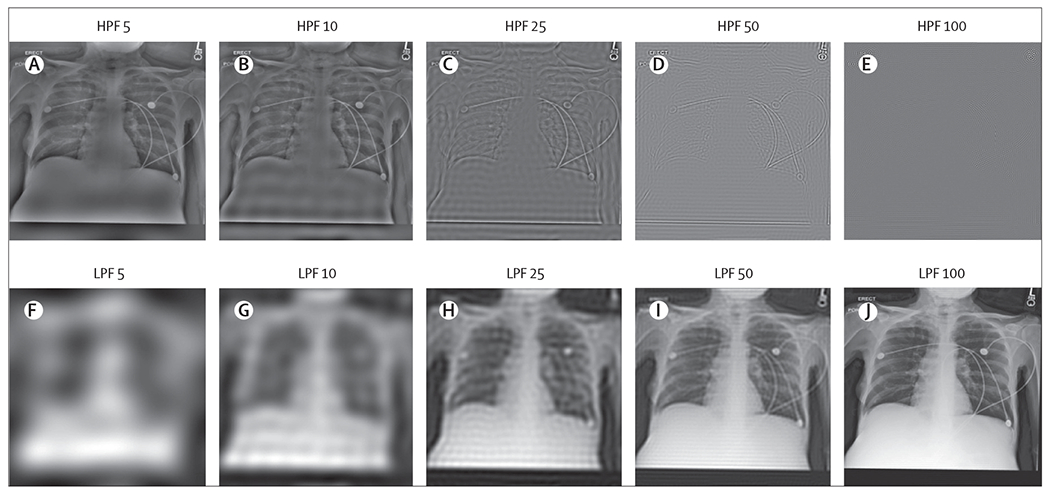
Samples of the images after low-pass filters and high-pass filters in MXR dataset HPF=high-pass filtering. LPF=low-pass filtering. MXR=MIMIC-CXR dataset.

We also examined whether race information persisted in all spectral ranges and in the presence of highly degraded images. As shown in figure 1, we tested the effect on model performance of adding a low-pass filter and a high-pass filter for various diameters in the MXR dataset, and show samples of the transformed images in figure 2. The addition of a low-pass filter resulted in significantly degraded performance at around diameter ten, which corresponded to high levels of visual degradation. A high performance (up to diameter 100) in the absence of discernible anatomical features was maintained with the addition of a high-pass filter (ie, model performance was maintained despite extreme degradation of the image visually). Further experiments that used band-pass and notch filtering are reported in the appendix (pp 25–26), with the transformed images visualised also given in the appendix (pp 7–8).

The AUC of various image resolutions, from 1 pixel resolution to 320 × 320 images in the MXR dataset, are shown in the appendix (p 12). For images at 160 × 160 resolution or higher, AUC values were >0·95. There was a reduction in performance for images below this resolution, which demonstrates that race information persisted more than random chance even for resolutions as small as 4 × 4 (appendix p 28). Similar results were observed for the perturbed images, with AUC values of 0·74 to 0·80 for the noisy images and 0·64 to 0·72 for the blurred images (appendix p 29).

Concerning whether race information was localised to a specific anatomical region or body segment, using data from multiple experiments from several datasets, there was no evidence of a clear contribution of any anatomical regions or body segments on race identity. Models tested on non-lung segmentations of images were better able to identify race compared with models tested on lung segmentations, but segmented predictions were lower than the original image predictions (appendix p 29). Therefore, the race information utilised by artificial intelligence was likely to be determined from a combination of information from all image segments, including both lung and non-lung segments. Similar findings were observed in slice-wise analysis of CT scans. Occluding the image regions identified by saliency maps (appendix p 9) caused a decrease in AUC values in race identification but still led to AUC values ≥0·67 (appendix p 29).

Race prediction was robust to the removal of any particular patch from images in the MXR dataset, indicating that race information was not localised within a specific part of the 3 × 3 grid (appendix p 30). We observed that there are parts of the image with little race information (appendix p 30). However, in most cases, using only one ninth of the image was sufficient to obtain prediction performance that was almost identical to using the entire image (appendix p 30).

Race prediction performance was also robust across models trained on single equipment and single hospital location on the chest x-ray and mammogram datasets (appendix pp 30–31). We observed a decrease in performance (although the outputs were better than random) on the digitised chest x-ray in the CheXphoto dataset compared with the digital CXP dataset, implying that some signal still persisted with different image acquisitions (appendix p 31).

## Discussion

In this modelling study, which used both private and public datasets, we found that deep learning models can accurately predict the self-reported race of patients from medical images alone. This finding is striking as this task is generally not understood to be possible for human experts. We also showed that the ability of deep models to predict race was generalised across different clinical environments, medical imaging modalities, and patient populations, suggesting that these models do not rely on local idiosyncratic differences in how imaging studies are conducted for patients with different racial identities. Beyond these findings, in two of the datasets (MXR and CXP) analysed, all patients were imaged in the same locations and with the same processes, presumably independently of race.

We also provide evidence that disease distribution and body habitus of patients in the CXP, MXR, and EMX datasets were not strongly predictive of racial group, implying that the deep learning models were not relying on these features alone. Although an aggregation of these and other features could be partially responsible for the ability of AI models to detect racial identity in medical images, we could not identify any specific image-based covariates that could explain the high recognition performance presented here.

Our findings conflict with data from Jabbour and colleagues’ study,^34^ which measured the extent to which models learned potentially sensitive attributes (eg, age, race, and BMI) from an institutional dataset (the AHRF dataset) of 1296 patient chest x-rays. Their findings led to an AUC value of 0·66 (0·54–0·79). Possible explanations for this discrepant performance compared with our experiment could be due to the use of transfer learning in Jabbour and colleagues’ study, in which the MXR and CXP datasets were used for initial training, and the final layers were fine-tuned on the AHRF dataset. This possible contamination in the dataset might have degraded performance due to label misalignment. We do not have access to the AHRF dataset for further external validation and Jabbour and colleagues did not extend their experiments to MXR and CXP datasets.

The results of the low-pass filter and high-pass filter experiments done in our study suggest that features relevant to the recognition of racial identity were present throughout the image frequency spectrum. Models trained on low-pass filtered images maintained high performance even for highly degraded images. More strikingly, models that were trained on high-pass filtered images maintained performance well beyond the point that the degraded images contained no recognisable structures; to the human coauthors and radiologists it was not clear that the image was an x-ray at all. Furthermore, experiments that were involved in patch-based training, slice-based error analysis, and saliency mapping were non-contributory: no specific regions of the images consistently informed race recognition decisions. Overall, we were unable to isolate specific image features that were responsible for the recognition of racial identity in medical images, either by spatial location, in the frequency domain, or that were caused by common anatomic and phenotype confounders associated with racial identity.

Although the ability to accurately detect self-reported race from highly degraded x-ray images is not meaningful on its own, this ability is important in the larger sociotechnical context that AI models operate in for medical imaging. One commonly proposed method to mitigate the known disparity in AI model performance is through the selective removal of features that encode sensitive attributes to make AI models “colorblind”.^35^ Although this approach has already been criticised as being ineffective, or even harmful in some circumstances,^36^ our work suggests that such an approach could be impossible in medical imaging because racial identity information appears to be incredibly difficult to isolate. The ability to detect race was not mitigated by any reasonable reduction in resolution or by the addition of noise, nor by frequency spectrum filtering or patch-based masking. Even ignoring the question of whether these approaches were beneficial, it seems plausible that technical solutions along these lines are unlikely to succeed and that strategies designed to detect racial bias,^37^ paired with the intentional design of models to equalise racial outcomes,^38^ should be considered to be the default approach to optimise the safety and fairness of AI in this context. The regulatory environment in particular, while evolving, has not yet produced strong processes to guard against unexpected racial recognition by AI models; either to identify these capabilities in models or to mitigate the harms that might be caused.

There were several limitations to this work. Most importantly, we relied on self-reported race as the ground truth for our predictions. There has been extensive research into the association between self-reported race and genetic ancestry, which has shown that there is more genetic variation within races than between races, and that race is more a social construct than a biological construct.^24^ We note that in the context of racial discrimination and bias, the vector of harm is not genetic ancestry but the social and cultural construct that of racial identity, which we have defined as the combination of external perceptions and self-identification of race. Indeed, biased decisions are not informed by genetic ancestry information, which is not directly available to medical decision makers in almost any plausible scenario. As such, self-reported race should be considered a strong proxy for racial identity.

Our study was also limited by the availability of racial identity labels and the small cohorts of patients from many racial identity categories. As such, we focused on Asian, Black, and White patients, and excluded patient populations that were too small to adequately analyse (eg, Native American patients). Additionally, Hispanic patient populations were also excluded because of variations in how this population was recorded across datasets. Moreover, our experiments to exclude bone density involved brightness clipping at 60% and evaluating average body tissue pixels, with no methods to evaluate if there was residual bone tissue that remained on the images. Future work could look at isolating different signals before image reconstruction.

We finally note that this work did not establish new disparities in AI model performance by race. Our study was instead informed by previously published literature that has shown disparities in some of the tasks we investigated.^10,39^ The combination of reported disparities and the findings of this study suggest that the strong capacity of models to recognise race in medical images could lead to patient harm. In other words, AI models can not only predict the patients’ race from their medical images, but appear to make use of this capability to produce different health outcomes for members of different racial groups.

To conclude, our study showed that medical AI systems can easily learn to recognise self-reported racial identity from medical images, and that this capability is extremely difficult to isolate. We found that patient racial identity was readily learnable from medical imaging data alone, and could be generalised to external environments and across multiple imaging modalities. We strongly recommend that all developers, regulators, and users who are involved in medical image analysis consider the use of deep learning models with extreme caution as such information could be misused to perpetuate or even worsen the well documented racial disparities that exist in medical practice. Our findings indicate that future AI medical imaging work should emphasise explicit model performance audits on the basis of racial identity, sex, and age, and that medical imaging datasets should include the self-reported race of patients when possible to allow for further investigation and research into the human-hidden but model-decipherable information related to racial identity that these images appear to contain.

## Supplementary Material

1

## Figures and Tables

**Table 1:** Summary of datasets used for race prediction experiments

	MXR	CXP	EMX	NLST	RSPECT (Stanford subset)	EM-CT	DHA	EM-Mammo	EM-CS
Data type	Chest x-ray	Chest x-ray	Chest x-ray	Chest CT	Chest CT (PE protocol)	Chest CT	Digital radiography x-ray	Breast mammograms	Lateral c-spine x-ray

Number of patients (number of images)	53073 (228 915)	65400 (223 414)	90518 (227 872)	512 (198 475)	254 (72 329)	560 (187 513)	691 (691)	27160 (86 669)	997 (10 358)

Sex									
Female	27532 (51·9%)	29090 (44·5%)	48477 (53·6%)	184 (36·0%)	135 (53·1%)	286 (51·1%)	400 (49·2%)	27160 (100%)	535 (53·7%)
Male	25541 (48·1%)	36310 (55·5%)	42041 (46·4%)	328 (64·0%)	119 (46·9%)	274 (48·9%)	391 (56·6%)	0	462 (46·3%)

Race									
Black	8957 (16·9%)	3147 (4·8%)	42373 (46·8%)	241 (47·1%)	23 (9·1%)	403 (72·0%)	333 (48·2%)	13696 (50·4%)	247 (24·8%)
Asian	1935 (3·6%)	7096 (10·8%)	3293 (3·6%)	0	0	0	0	0	0
White	34035 (64·1%)	36765 (56·2%)	38071 (42·1%)	271 (53·0%)	231 (90·9%)	157 (28·0%)	358 (51·8%)	13464 (49·6%)	750 (75·2%)
Unknown	8146 (15·3%)	18420 (28·2%)	6781 (7·5%)	0	0	0	0	0	0

Dataset split									
Training, %	60·0%	60·0%	75·0%	78·0%	0	0	70·0%	60·0%	80·0%
Validation, %	10·0%	10·0%	12·5%	10·0%	0	0	10·0%	20·0%	10·0%
Test, %	30·0%	30·0%	12·5%	12·0%	100·0%	100·0%	20·0%	20·0%	10·0%

CXP=CheXpert dataset. DHA=Digital Hand Atlas. EM-CS=Emory Cervical Spine radiograph dataset. EM-CT=Emory Chest CT dataset. EM-Mammo=Emory Mammogram dataset. EMX=Emory chest x-ray dataset. MXR=MIMIC-CXR dataset. NLST=National Lung Cancer Screening Trial dataset. RSPECT=RSNA Pulmonary Embolism CT dataset.

**Table 2:** Summary of experiments conducted to investigate mechanisms of race detection in Black patients

	Area under the receiver operating characteristics curve
**Race detection in radiology imaging**	

Chest x-ray (internal validation)*	
MXR (Resnet34, Densenet121)	0·97, 0·94
CXP (Resnet 34)	0·98
EMX (Resnet34, Densenet121, EfficientNet-B0)	0·98, 0·97, 0·99
Chest x-ray (external validation)*	
MXR to CXP, MXR to EMX	0·97, 0·97
CXP to EMX, CXP to MXR	0·97, 0·96
EMX to MXR, EMX to CXP	0·98, 0·98
Chest x-ray (comparison of models)†	
MXR, CXP, EMX	Multiple results (appendix p 26)
CT chest (internal validation)*	
NLST (slice, study)	0·92, 0·96
CT chest (external validation)*	
NLST to EM-CT (slice, study)	0·80, 0·87
NLST to RSPECT (slice, study)	0·83, 0·90
Limb x-ray (internal validation)*	
DHA	0·91
Mammography*	
EM-Mammo (image, study)	0·78, 0·81
Cervical spine x-ray*	
EM-CS	0·92

**Experiments on anatomic and phenotypic confounders**	

BMI*	
CXP	0·55, 0·52
Image-based race detection stratified by BMI†	
EMX, MXR	Multiple results (appendix p 24)
Breast density*	
EM-Mammo	0·54
Breast density and age*	
EM-Mammo	0·61
Disease distribution*	
MXR, CXP	0·61, 0·57
Image-based race detection for the no finding class*	
MXR	0·94
Model prediction after training on dataset with equal disease distribution†	
MXR	0·75
Removal of bone density features*	
MXR, CXP	0·96, 0·94
Impact of average pixel thresholds†	
MXR	0·50
Impact of age†	
MXR	Multiple results (appendix p 27)
Impact of patient sex†	
MXR	Multiple results (appendix p 28)
Combination of age, sex, disease, and body habitus*	
EMX (logistic regression model, random forest classifier, XGBoost model)	0·65, 0·64, 0.64

**Experiments to evaluate the mechanism of race detection**	

Frequency domain filtering	
High-pass filtering*	
MXR	Multiple results (appendix p 26)
Low-pass filtering*	
MXR	Multiple results (appendix p 26)
Notch filtering†	
MXR	Multiple results (appendix p 26)
Band-pass filtering†	
MXR	Multiple results (appendix p 25)
Image resolution and quality*	
MXR	Multiple results (appendix p 28)
Anatomical localisation	
Lung segmentation experiments†	
MXR	Multiple results (appendix p 29)
Saliency maps†	
MXR, CXP, EMX, NLST, DHA, EM-Mammo, EM-CS	Multiple results (appendix pp 13–18)
Occlusion experiments†	
MXR	Multiple results (appendix p 30)
Patch-based training*	
MXR	Multiple results (appendix p 30)
Image acquisition differences†	
EMX, EM-Mammo, ChexPhoto	Multiple results (appendix p 31)

BMI=body-mass index. CXP=CheXpert dataset. DHA=Digital Hand Atlas. EM-CS=Emory Cervical Spine radiograph dataset. EM-CT=Emory Chest CT dataset. EM-Mammo=Emory Mammogram dataset. EMX=Emory CXR dataset. MXR=MIMIC-CXR dataset. NLST=National Lung Cancer Screening Trial dataset. RSPECT=RSNA Pulmonary Embolism CT dataset.

* Results located in main text.

† Results located in the appendix.

**Table 3:** Performance of deep learning models to detect race from chest x-rays

	Area under the receiver operating characteristics curve value for race classification		
	Asian (95% CI)	Black (95% CI)	White (95% CI)
**Primary race detection in chest x-ray imaging**			

MXR Resnet34	0·986 (0·984–0·988)	0·982 (0·981–0·983)	0·981 (0·979–0·982)
CXP Resnet34	0·981 (0·979–0·983)	0·980 (0·977–0·983)	0·980 (0·978–0·981)
EMX Resnet34	0·969 (0·961–0·976)	0·992 (0·991–0·994)	0·988 (0·986–0·989)

**External validation of race detection models in chest x-ray imaging**			

MXR Resnet34 to CXP	0·947 (0·944–0·951)	0·962 (0·957–0·966)	0·948 (0·945–0·951)
MXR Resnet34 to EMX	0·914 (0·899–0·928)	0·983 (0·981–0·985)	0·975 (0·973–0·978)
CXP Resnet34 to MXR	0·974 (0·971–0·977)	0·955 (0·952–0·957)	0·956 (0·954–0·958)
CXP Resnet34 to EMX	0·915 (0·901–0·929)	0·968 (0·965–0·971)	0·954 (0·951–0·958)
EMX Resnet34 to MXR	0·966 (0·962–0·969)	0·970 (0·968–0·972)	0·964 (0·962–0·965)
EMX Resnet34 to CXP	0·949 (0·946–0·952)	0·973 (0·970–0·977)	0·947 (0·945–0·950)

**Race detection in non-chest x-ray imaging modalities: binary race detection (Black or White)**			

NLST	0·92 (slice; 0·910–0·918), 0·96 (study; 0·926–0·982)	··	··
NLST to EM-CT	0·80 (slice; 0·796–0·800), 0·87 (study; 0·829–0·904)	··	··
NLST to RSPECT	0·83 (slice; 0·825–0·834), 0·90 (study; 0·836–0·958)	··	··
EM-Mammo	0·78 (slice; 0·773–0·786), 0·81 (study; 0·794–0·818)	··	··
EM-CS	0·913 (0·892–0·931)	··	··
DHA	0·87 (0·752–0·894)	··	··

Values reflect the area under the receiver operating characteristics curve for each model on the test set per slice and per study (by averaging the predictions across all slices). CXP=CheXpert dataset. DHA=Digital Hand Atlas. EM-CS=Emory Cervical Spine radiograph dataset. EM-CT=Emory Chest CT dataset. EM-Mammo=Emory Mammogram dataset. EMX=Emory CXR dataset. MXR=MIMIC-CXR dataset. NLST=National Lung Cancer Screening Trial dataset. RSPECT=RSNA Pulmonary Embolism CT dataset.

## Data Availability

The MIMIC-CXR dataset, CheXpert dataset, National lung cancer screening trial, RSNA Pulmonary Embolism CT, and the Digital Hand Atlas are all publicly available. The Emory University datasets (Emory CXR, Emory Chest CT, Emory Cervical Spine, and Emory Mammogram) are available on request after signing a data use agreement. All code is available at https://github.com/Emory-HITI/AI-Vengers.

## Research in context

### Evidence before this study

We used three different search engines to do our review. For PubMed, we used the following search terms: “(((disparity OR bias OR fairness) AND (classification)) AND (x-ray OR mammography)) AND (machine learning [MeSH Terms]).” For IEEE Xplore, we used the following search terms: “((disparity OR bias OR fairness) AND (mammography OR x-ray) AND (machine learning))”. For ACM, we used the following search terms: “[Abstract: mammography x-ray] AND [Abstract: classification prediction] AND [All: disparity fairness]”. All queries were limited to dates between Jan 1, 2010, and Dec 31, 2020. We included any studies that were published in English, focused on medical images, and that were original research. We also reviewed commentaries and opinion articles. We excluded articles that were not written in English or that were outside of the medical imaging domain. To our knowledge, there is no published meta-analysis or systematic review on this topic. Most published papers focused on measuring disparities in tabular health data without much emphasis on imaging-based approaches.

Although previous work has shown the existence of racial disparities, the mechanism for these differences in medical imaging is, to the best of our knowledge, unexplored. Pierson and colleagues noted that an artificial intelligence (AI) model that was designed to predict severity of osteoarthritis using knee x-rays could not identify the race of the patients. Yi and colleagues conducted a forensics evaluation on chest x-rays and found that AI algorithms could predict sex, distinguish between adult and paediatric patients, and differentiate between US and Chinese patients. In ophthalmology, retinal scan images have been used to predict sex, age, and cardiac markers (eg, hypertension and smoking status). We found few published studies that explicitly targeted the recognition of racial identity from medical images, possibly because radiologists do not routinely have access to, nor rely on, demographic information (eg, race) for diagnostic tasks in clinical practice.

### Added value of this study

In this study, we investigated a large number of publicly and privately available large-scale medical imaging datasets and found that self-reported race is accurately predictable by AI models trained with medical image pixel data alone as model inputs. First, we showed that AI models are able to predict race across multiple imaging modalities, various datasets, and diverse clinical tasks. This high level of performance persisted during external validation of these models across a range of academic centres and patient populations in the USA, as well as when the models were optimised to do clinically motivated tasks. Second, we conducted ablations that showed that this detection was not due to trivial proxies, such as body habitus, age, tissue density, or other potential imaging confounders for race (eg, underlying disease distribution in the population). Finally, we showed that the features learned appear to involve all regions of the image and frequency spectrum, suggesting the efforts to control this behaviour when it is undesirable will be challenging and demand further study.

### Implications of all the available evidence

In our study, we emphasise that the ability of AI to predict racial identity is itself not the issue of importance, but rather that this capability is readily learned and therefore is likely to be present in many medical image analysis models, providing a direct vector for the reproduction or exacerbation of the racial disparities that already exist in medical practice. This risk is compounded by the fact that human experts cannot similarly identify racial identity from medical images, meaning that human oversight of AI models is of limited use to recognise and mitigate this problem. This issue creates an enormous risk for all model deployments in medical imaging: if an AI model relies on its ability to detect racial identity to make medical decisions, but in doing so produced race-specific errors, clinical radiologists (who do not typically have access to racial demographic information) would not be able to tell, thereby possibly leading to errors in health-care decision processes.
