# Supplementary material for: AI recognition of patient race in medical imaging: a modelling study
Source: Lancet Digit Health. Author manuscript; Available in PMC 2022 Nov 11. (PMC9650160; doi:10.1016/S2589-7500(22)00063-2)
Supplement: 1 [file NIHMS1810682-supplement-1.pdf]

THE LANCET
Digital Health

Supplementary appendix

This appendix formed part of the original submission and has been peer reviewed. We post it as supplied by the authors.

Supplement to: Gichoya JW, Banerjee I, Bhimireddy AR, et al. AI recognition of patient race in medical imaging: a modelling study. *Lancet Digit Health* 2022; published online May 11. https://doi.org/10.1016/S2589-7500(22)00063-2.

Supplementary Appendix

Table of Contents

Methods	2
Figure S1	8
Figure S2	8
Figure S3	9
Figure S4	9
Figure S5	10
Figure S6	10
Figure S7	11
Figure S8	12
Figure S9	13
Figure S10	15
Figure S11	16
Figure S12	18
Figure S13	19
Figure S14	20
Figure S15	20
Figure S16	21
Table S1	22
Table S2	24
Table S3	24
Table S4	24
Table S5	25
Table S6	25
Table S7	25
Table S8	26
Table S9	26
Table S10	27
Table S11	27
Table S12	28
Table S13	28
Table S14	28
Table S15	29
Table S16	29
Table S17	29
Table S18	30
Table S19	30
Table S20	30
Table S21	30
Table S22	31
Table S23	31
References	32

Methods

Datasets

For CXR data, we utilised MIMIC-CXR (MXR)(1), CheXpert (CXP)(2), and Emory-CXR (EMX) obtained from Emory Hospital. For limb x-ray imaging, we used the digital hand atlas (DHA) dataset(3). For CT imaging, the model was trained on a subset of the National Lung Screening Trial (NLST)(4) dataset and externally validated on the Stanford subset of the RSNA-STR Pulmonary Embolism CT (RSPECT) dataset (5) for which we were able to separately obtain the race labels, and a CT chest dataset from Emory Hospital (EM-CT). A screening mammogram dataset (EM-Mammo) and a cervical spine x-ray dataset (EM-CS) were acquired from Emory University Hospital. Each dataset included images, disease class labels, and race/ethnicity labels including Black/African American and White. Asian labels were available in some datasets (MXR, CXP, EMX and DHA) and were utilised when available and the population prevalence was above 1%. Hispanic/Latino labels were only available in some datasets and were coded heterogeneously, so patients with these labels were excluded from analysis.

Ethical approval was obtained for the Emory datasets from the Emory Institutional Review Board (Chest x-ray - IRB00091978 ; Mammograms - STUDY00000673 ; Cervical hardware - IRB00111139 ; CT chest STUDY00000506). Use of the NLST dataset was approved under project NLST-782. The data in MXR has been previously de-identified, and approved by the institutional review boards of Massachusetts Institute of Technology (No. 0403000206) and Beth Israel Deaconess Medical Center (2001-P-001699/14) for research. The CXP and RSPECT (Stanford subset) datasets were de-identified per Stanford institutional guidelines and deemed non-human subjects research data and therefore institutional IRB was waived per policy. Research use of the data set was in compliance with the Stanford data use agreement.

General model training details

Model Architectures:
The details of model settings are listed in the Supplemental Table S1 while datasets splits are summarized in Table 1 in the main manuscript. The CNN model architectures were selected based on dataset size and task complexity. CXR race classification models were trained using Resnet34 (6), Densenet121 (7) and EfficientNetB0 (8) architectures with pre-trained weights from ImageNet. We trained a Resnet50 (6) baseline model on the digital hand atlas, Resnet34 (6) model on cervical spine radiographs, and EfficientNetB2 (8) model on the mammogram images. A Densenet121 (7) model was trained on the NLST chest CT images and externally validated on the RSPECT and EM-CT datasets.

Model Parameters:
Images were resized to sizes between 224 and 320. A random seed of 2021 and bootstrap of 1000 was used for all experiments. Hyperparameters including random horizontal flip, random 15 degree rotation, and random zoom of ±10% were applied during training. Adam optimization algorithm was chosen with a categorical cross-entropy loss function and a starting learning rate of 1e-3 that decreased by a factor of ten after two consecutive epochs without improvement in overall validation loss. We used a batch size of 256. Importantly, these experiments were performed using the standard model implementations included in the

public Keras package distributed with the Tensorflow library(9), one of the most popular python libraries for CNN model development.

Definition of ROC-AUC as the evaluation metric:
In our analysis and result reporting, we use the ROC-AUC metric(10). A ROC curve (Receiver Operating Characteristics) is a graph showing the performance of a classification model at all classification thresholds. The curve is plotted using two parameters - True Positive Rate as the x-axis and False Positive Rate as the y-axis. AUC stands for - Area Under the ROC Curve and measures the two-dimensional area underneath the ROC curve.

Justification for use of confidence intervals and not performing calibration assessment:
Calibration assessment was not performed because the objective is not to generate a probabilistic estimate from the model but to present the performance of the model for race discrimination. According to TRIPOD (Transparent Reporting of a multivariable prediction model for Individual Prognosis Or Diagnosis) guidelines(11,12), calibration assessment is needed for prediction modeling studies for healthcare. Instead, we calculated the Confidence intervals (CI) around the ROC-AUC to estimate the upper and lower limit of the classifier performance. It is a standard way of quantifying the uncertainty of an estimate generated by a machine learning model and provides the error rate. Given the tighter CI, we can conclude the models are generating highly precise labels with low error margin(13).

Code availability:
All code for the various experiments is available with an open-source license at https://github.com/Emory-HITI/AI-Vengers.

Chest CT image preprocessing

The chest CT images were preprocessed by standardizing the rescale intercept value to 1024 and normalizing the pixel values of the images by dividing by 3000. In addition, we also dropped images that had abnormal pixel values for air. This was determined by selecting an empty patch on the image and calculating the minimum pixel value within that patch. If the value was outside of the interval [-30, 30] then the image was dropped.

Specific model training details

A: RACE DETECTION IN RADIOLOGY IMAGING

A1. Primary race detection

We trained and evaluated three models on the three CXR datasets (Table 1 - main manuscript) to predict if the patient's self-reported race was Black, White or Asian. Model training details are summarized in the Supplemental Table S1. Given no difference in performance of various architectures on race prediction, we selected the Resnet34(6) model for external validation between the CXR datasets. Detection performance was characterised with ROC-AUC with a one-vs-rest approach for each racial group.

A2. Race detection in non-CXR imaging

Due to limited availability of the Asian class in non CXR datasets, we performed a binary classification to identify racial identity in Black patients versus White patients on the digital hand atlas, cervical spine radiographs, NLST chest CT and mammogram images (Table S1). The chest CT model was externally validated on the RSPECT and EM-CT datasets. Detection performance was characterised with ROC-AUC with a one-vs-rest approach for each racial group. For multislice studies, predictions were made at the slice level, with aggregated performance at the study level.

B: EXPERIMENTS ON ANATOMICAL AND PHENOTYPE CONFOUNDERS

B1. Race detection using *body habitus*

We assessed the relationship between *body habitus* (obtained from the recorded body mass index - BMI) and race for Black and White patients in several datasets, and with several different methods. First, we tested the correlation between BMI and race in the CXP dataset by training a logistic regression model(14) to predict race from BMI. Secondly, we performed stratified training and testing on the MXR dataset classified into four standard BMI groups (Table S2). Thirdly, we performed subset analysis of a trained race detection model on the EMX dataset, reporting the performance of the model at differentiating Asian, Black and White patients in four BMI groups (Table S4).

B2. Tissue density analysis on mammograms

We assessed the relationship between breast density and race for Black and White patients in the EM-Mammo dataset (Table S5). We trained two distinct multi-class logistic regression models (15) (one-vs-rest) to predict patient race based on the breast density and age.

B3. Race detection using disease labels

To evaluate the possibility that features related to disease distribution were responsible for the ability of models to detect race from CXRs, we trained models to predict race from the disease label data (i.e., without the images) on the MXR and CXP datasets using all available labels (14 labels, including the “no finding” and “support devices” labels). The disease labels for the MXR (1) and CXP (2) datasets have been published previously. We split each dataset into a 70% training, 30% test set. We trained an XGBoost(16) classifier, a L1-regularized logistic regression, and a random forest classifier to predict the patient’s race. We tuned hyperparameters (maximum depth for the tree-based models, and the regularization strength of logistic regression) using 5-fold cross validation on the training set. We present stratified results to show model performance on the test for the “no finding” class for the MXR and CXP datasets.

B4. Race detection using bone density

We removed bone density information within MXR and CXP images by clipping bright pixels to 60% intensity. Sample images are shown in Figure S1. Densenet-121 models were trained on the brightness-clipped images.

We conducted a second experiment where we used the overall pixel intensity of CXRs on the MIMIC-CXR dataset as input for a simple classifier with single input and no hidden layers, and a softmax classifier to detect race Black and White patients. The following tissue pixel intensity thresholds were used

- Normal (0 - 255)
- Air (30 - 255)
- Fat (80 - 255)
- Soft Tissue (110 - 255)
- Bone (180 - 255)

These are known/established pixel intensities for various body tissues, and are established for the windowing levels for CT scans (17).

B5. Race detection using age and sex

We investigated whether there is a cumulative effect of societal bias that impacts patients' general health, which is then used by the models as a proxy for race. We specifically examined whether there is a dose-response effect of race detection as people age, i.e., if features related to an underlying systemic health inequity are a proxy for race, then this should be more obvious in older patients. We split patients in MXR into five age groups as summarized in Table S10 in the and trained a Densenet121 model as described in Table S1.

We performed a second similar experiment by splitting the MXR datasets into male and female summarized in Table S12 and trained a Densenet121 model as described in Table S1.

B6. Race detection using combination of age, sex, disease and body habitus

We selected a subset of data from the Emory dataset that contained all variables of age, sex, disease labels (all 14 CXR labels) and BMI (Total dataset size of 123,003 images). The data were split into 70 % for training and 30 % test dataset. Due to the low numbers of the Asian patients in this sub cohort, we trained a binary classifier of Black and White patients. All the variables were one hot encoded and logistic regression model, random forest and XGBoost models trained.

C: EXPERIMENTS TO EVALUATE THE MECHANISM OF RACE DETECTION

C1. Frequency-domain imaging features

Given the lack of reported racial anatomical differences in the radiology literature and the known capability of deep learning models to utilise subtle textural cues that humans cannot perceive (18,19), we investigated the relative contributions of large-scale structural features and fine textural features by performing training and testing on datasets altered by filtering the frequency spectrum of the images.

Following the procedure outlined by previous work(18,20,21), we first transform each image into the frequency domain using a 2D Fourier transform. We then apply low-pass filtering (LPF) where we set all

frequency components outside a centered circle with diameter *d* to zero, and high-pass filtering (HPF), where all frequency components within a centered circle with diameter *d* are set to zero. We also test bandpass filtering (BF) and notch filtering (NF) and report these results in Figures S2 and S3 and Tables S7 and S8. All experiments were performed multiple times while varying the radius of the frequency spectrum filters.

After filtering, we applied the inverse Fourier transform on the filtered spectra to obtain an altered version of the original image and subsequently trained models on these perturbed datasets to observe the effect on the model's ability to predict race. These experiments were performed on the MXR dataset.

C2. Impact of image resolution and quality

To test whether race information was encoded in higher resolution images, we resized the MXR images into various resolutions and trained a Resnet34 model. To examine whether the image perturbations made an impact on race detection, we made the testing images in the MXR dataset noisy and blurred by adding gaussian noise (mean=0, variance=0.1) and applying a gaussian filter to them, respectively (Figure S4).

C3. Anatomical localisation

We investigated whether race information could be localized to a particular anatomical region or tissue by producing saliency maps for random cases for each task using the grad-cam methodology (22). Thereafter, five radiologists performed qualitative evaluation of these artefacts. We used the standard keras grad-cam implementation to generate saliency maps on the CXR datasets (Figure S9), digital hand atlas dataset (Figure S11), CT chest ((Figure S11), Emory Cervical spine radiographs (Figure S11) and mammogram datasets (Figure S11). For the CXR datasets, saliency maps were randomly generated from the test set for each race when correctly and incorrectly classified (Figure S9). The mammogram grad-cams were generated for each race and breast density classification (Figure S10).

We further evaluated the significance of the regions of interest as indicated by the saliency maps by masking out the region of interest in each MXR CXR heatmap as shown in Figure S5. We masked pixels with blue channels larger than 0.1 in the heatmap and then produced a minimum rectangle area to cover all masked pixels. The masked CXRs were used to test the model trained on original MXR images.
We also tested the performance of Densenet121 using CXR images consisting of lung and non-lung segmentations using an automatic segmentation algorithm (TernausNet) (23) on the MXR dataset, with manual checks on each image to exclude poorly segmented images (Figure S6). The numbers of segmented CXRs used for testing are 148, 382, and 200 for White, Black, and Asian patients respectively. This segmentation dataset will be released through the PhysioNet (https://physionet.org/) data repository. Details of model training can be found in Table S1.

We analyzed slice by slice results of the CT chest model demonstrating the distribution of errors by slice-location, to reveal whether any particular anatomical region (i.e., slices from the neck, upper chest, upper abdomen etc.) appear to be more useful for race detection.

C4. Patch-based training

We investigated whether race information can be isolated to specific patches within the chest x-ray images, for example, to exclude the possibility that hospital process features such as radiographic markers were responsible for the recognition of racial identity. On the MXR dataset, we split each image into nine 3x3 square cells of equal size (Figure S7). We experimented with training a race prediction model using two different approaches: (1) We select one of the nine patches, and completely remove all information from the patch by setting all pixels within the patch to zero and (2) We select one of the nine patches, scale it back to the size of the original image, and use only this patch for modeling. We show an example of patched images in Figure S7. We trained several networks for both approaches while varying the selected patch.

C5. Image acquisition differences

We extracted all CXR from a single hospital acquired on the Carestream portable CXR equipment, where we extracted 55,000 images. We trained a Densenet model on this dataset for race prediction (Model A). Thereafter, we tested the model on a test dataset composed of multiple hospitals and a mixture of CXRs obtained from the Carestream and GE imaging equipment.

We repeated a similar experiment on the mammogram dataset where we trained an EfficientNetB2 model on datasets obtained from single hospital locations and imaging equipment. Figure S13 shows the distribution of 2D mammogram imaging across various hospital locations by race, and Figure S14 shows distribution of a single image view by manufacturer.

Using the publicly available CheXphoto dataset(24), we selected approximately 6,000 CXR images that were individually displayed on a screen and then captured with a cell phone camera. These selected images were matched with their CheXpert metadata (Figure S15). A race classification model was then trained on these images. In addition, the same selected images from the original CheXpert dataset were also trained for race detection in order to compare performance differences.

Figure S1: Samples images in MXR with bone density information removed by clipping pixels at 60% brightness.

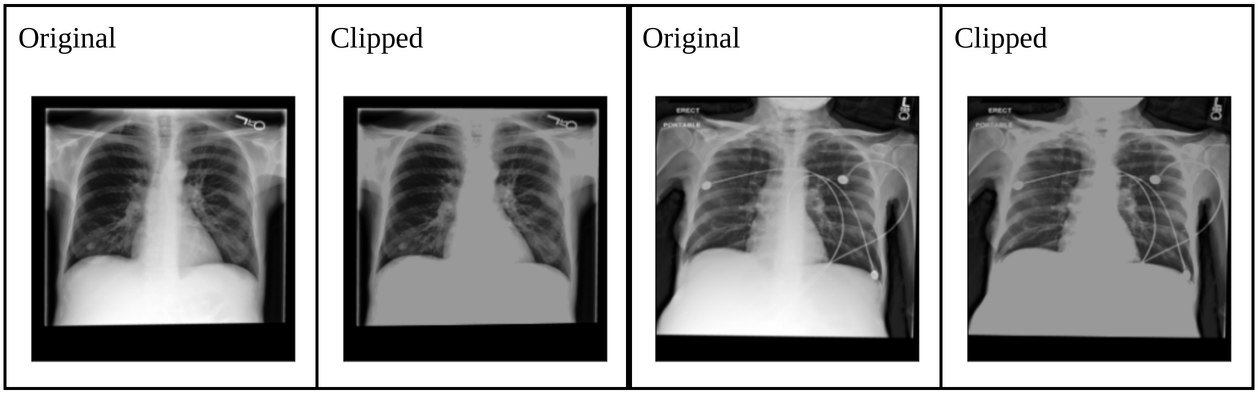

Figure S2: Transformed MXR images after bandpass filtering using various values of d_1 and d_2

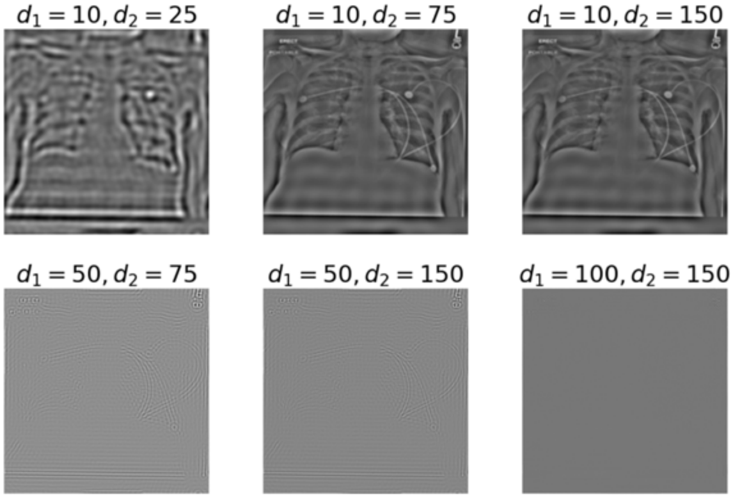

Figure S3: Transformed MXR images after notch filtering using various values of d_1 and d_2

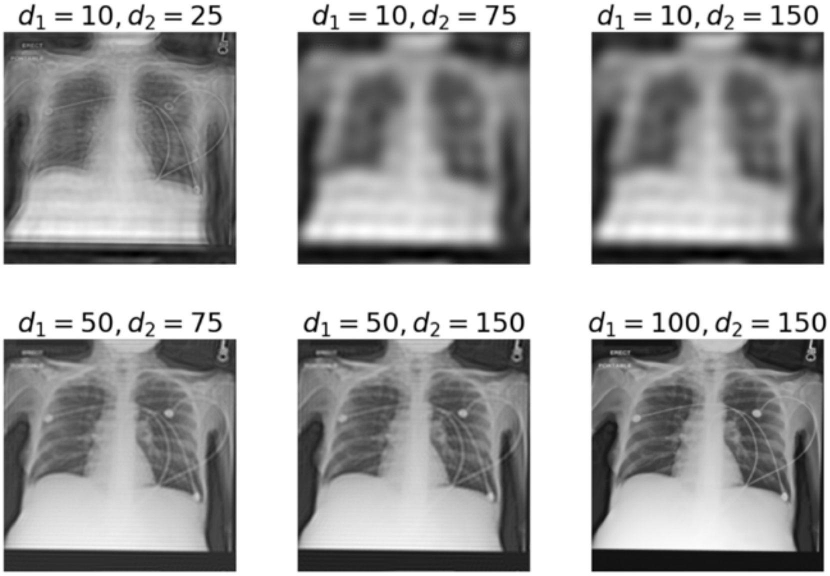

Figure S4: Examples of noisy (left) and blurred (right) images.

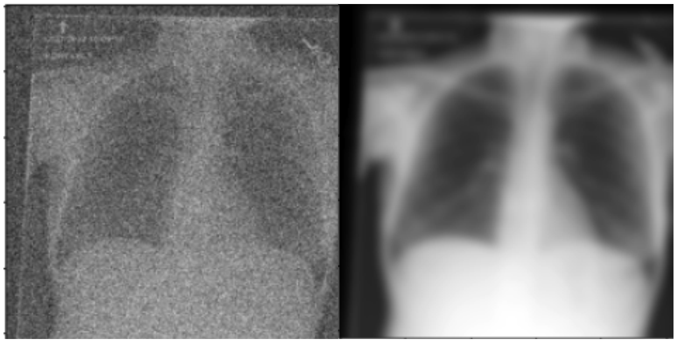

Figure S5: On the left image, there is a grad-cam saliency map showing the areas of highest probability for the race prediction model. On the right image, the pixels where the blue channels are > 0.1 are occluded with a rectangular mask.

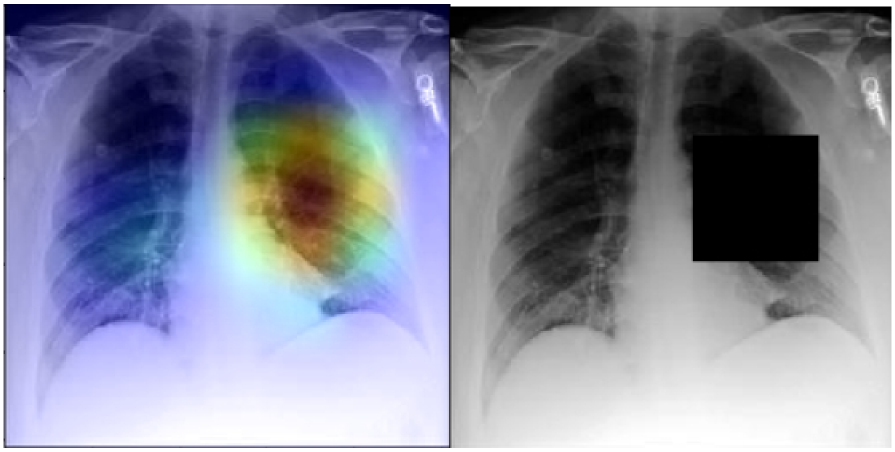

Figure S6: An example of lung segmentation from MXR. The original, non-lung segmented, and lung segmented images were used as the test data separately.

Original	Non-lung segmentation	Lung segmentation
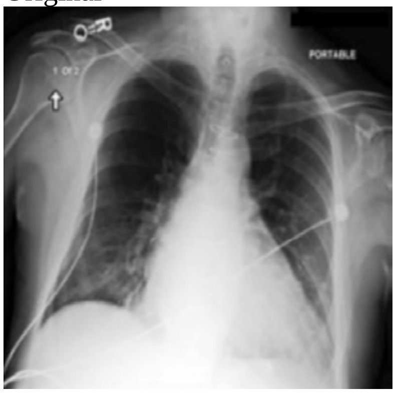	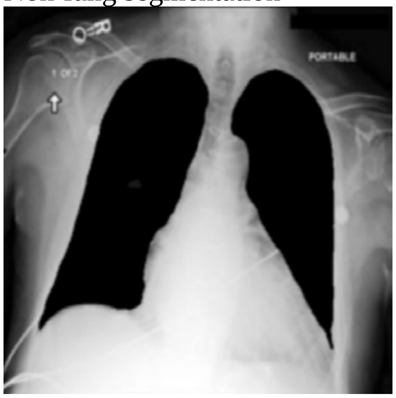	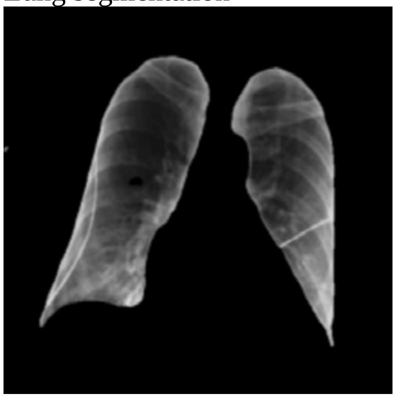

Figure S7: Sample images used for patch-based training. a) the original, unaltered image. b) the image after removing the patch located at quadrant (2, 1). c) training with only the patch located at quadrant (2, 1).

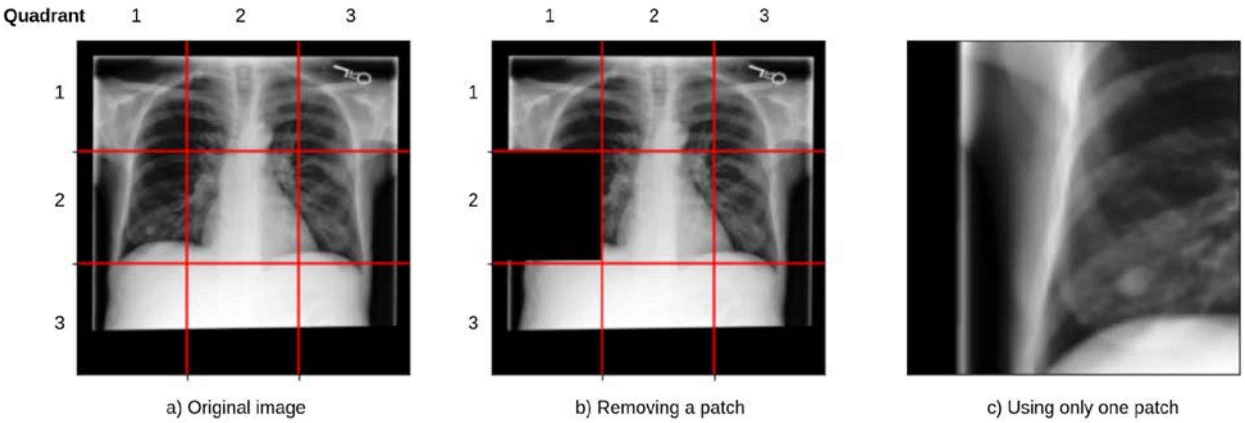

Figure S8: The performance of models trained and tested on various image resolutions from 1 pixel to 320x320 images for Asians, Blacks and Whites. High AUC values are maintained across various image resolutions. Zoomed plots of AUC predictions at lower image resolutions and corresponding appearance of CXR images.

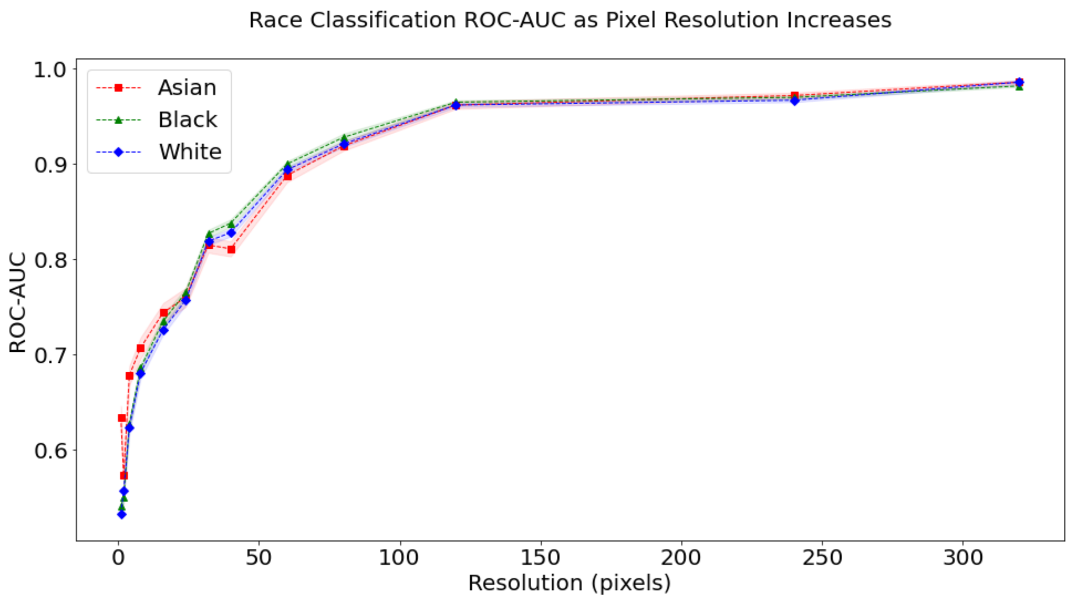

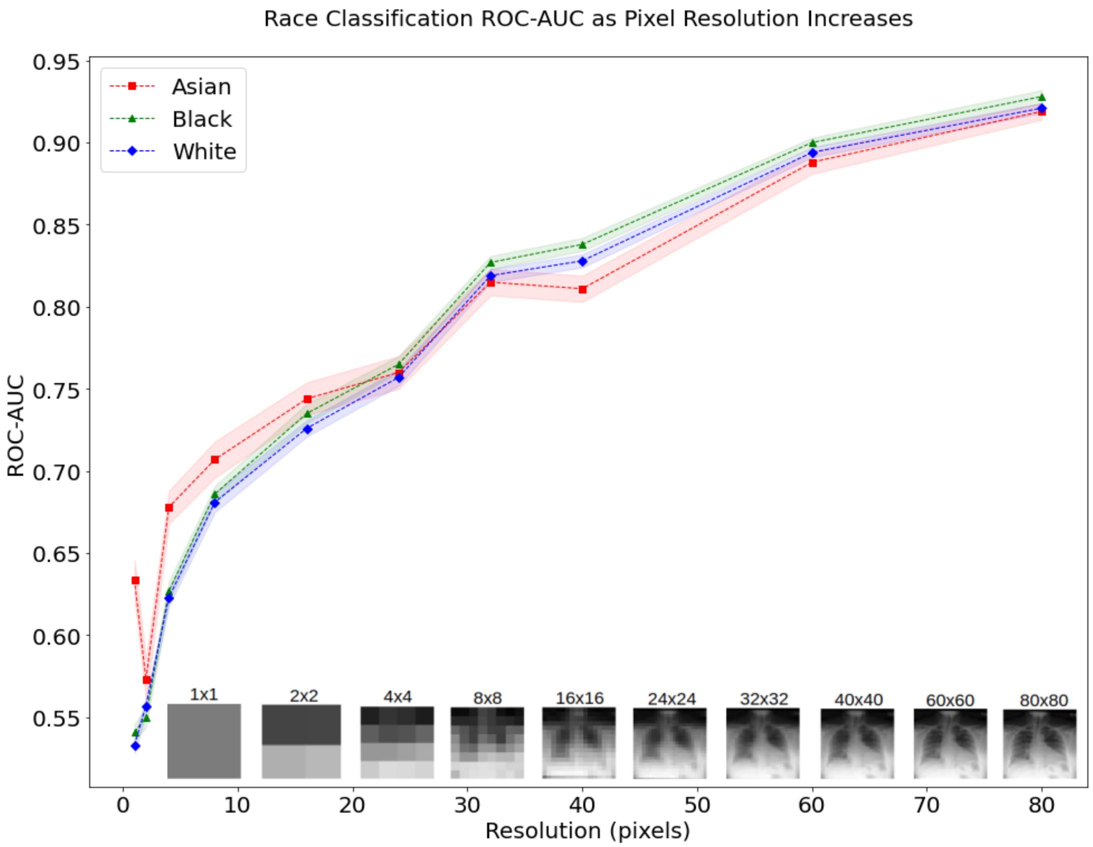

	Figure S9: Saliency maps for primary race detection for CXR images. The saliency maps were assessed qualitatively by all group members, across all tasks, including by members with radiology expertise. No consistent anatomical localisation was appreciated, and no anatomic structures appeared to be particularly salient to the decision making process.			
		Asian	Black	White
A1	Accurate primary race prediction	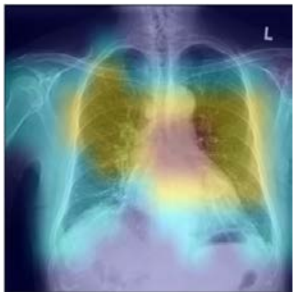	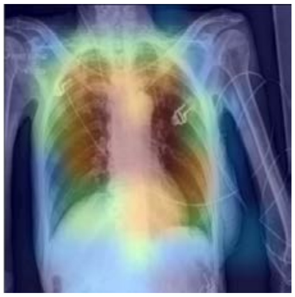	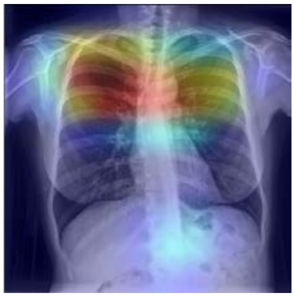
B3	Accurate primary race prediction from the “no finding” class label	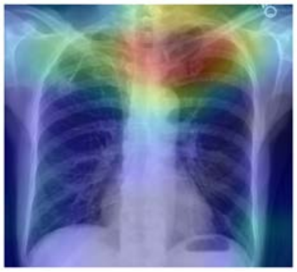	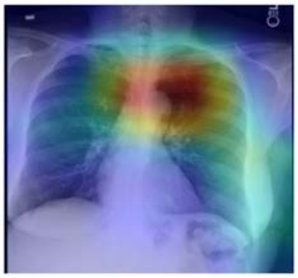	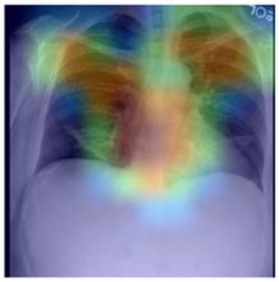
		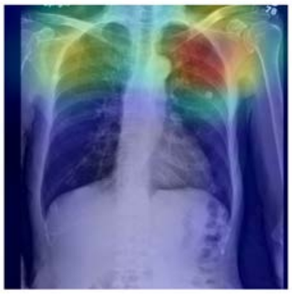	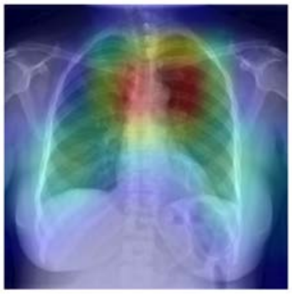	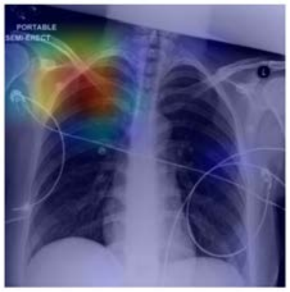
A1	Incorrectly classified race prediction	**Incorrectly predicted Asian**	**Incorrectly predicted Black**	**Incorrectly predicted White**
		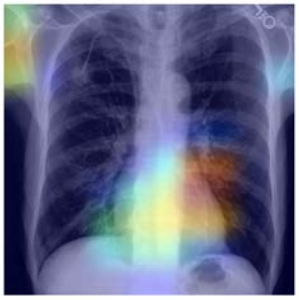	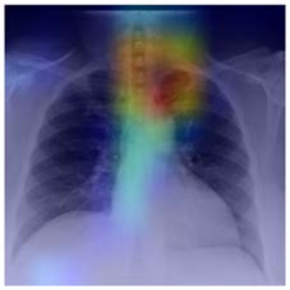	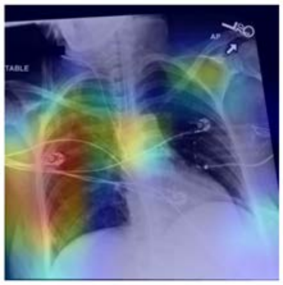 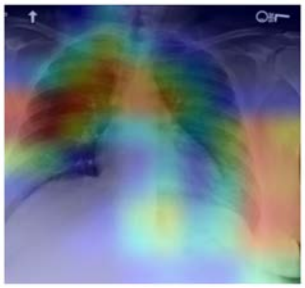

		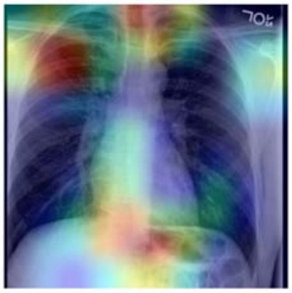<br>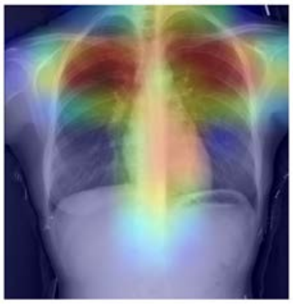	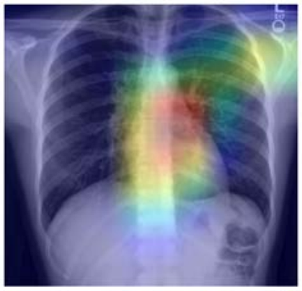<br>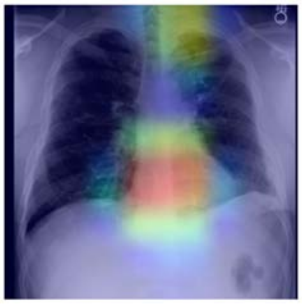	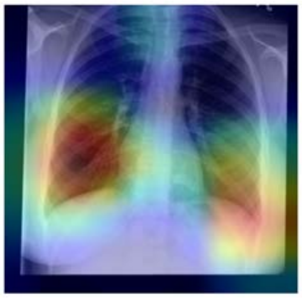

Figure S10: Generated saliency maps for Black and White patients across various breast density classes. Visual assessment by the team including four radiologists of a random sample of saliency maps did not produce any identifiable pattern that could explain race prediction.

Race/Breast Density	1 (Fatty)	2 (Scattered)	3 (Heterogeneous)	4 (Dense)
Original Image	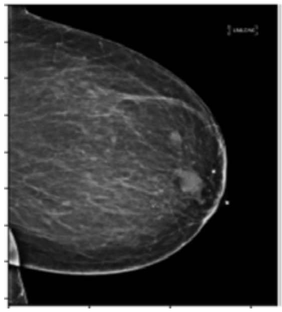	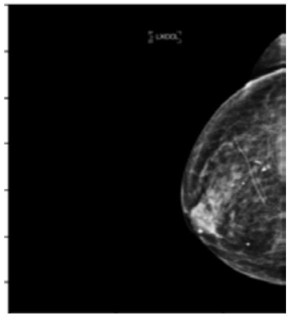	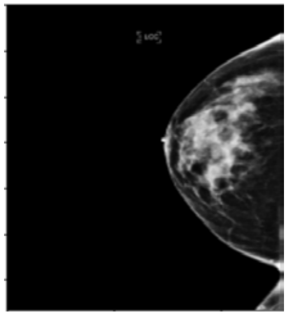	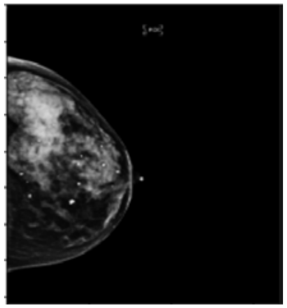
Black	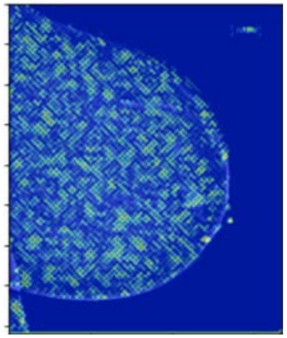	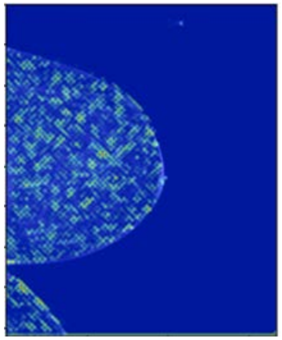	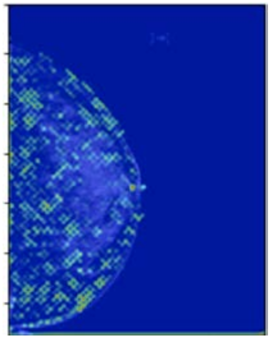	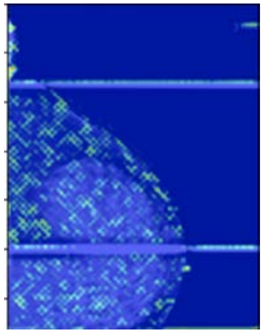
White	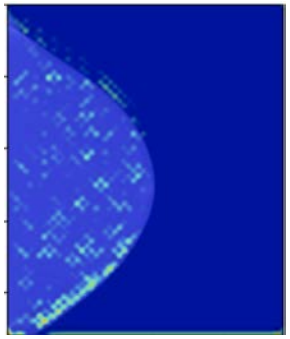	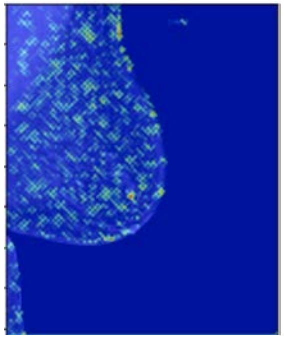	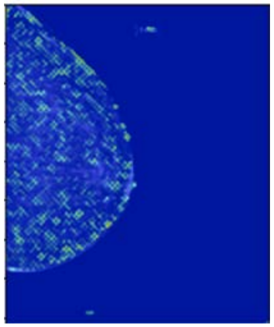	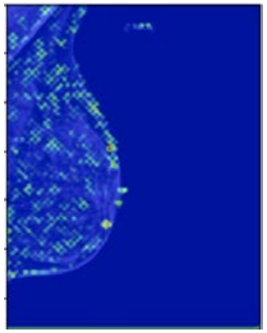

Figure S11: Saliency maps for primary race detection for non-CXR images. The saliency maps were assessed qualitatively by all group members, across all tasks, including by members with radiology expertise. No consistent anatomical localisation was appreciated, and no anatomic structures appeared to be particularly salient to the decision making process.

A2	Accurate primary race prediction - Digital Hand Atlas	Black	White
		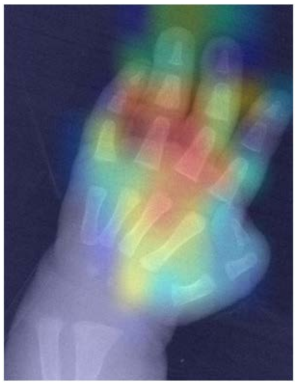	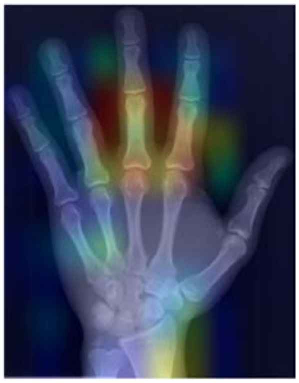
	Incorrectly classified race prediction - DHA	Black incorrectly classified as White	White incorrectly classified as Black
		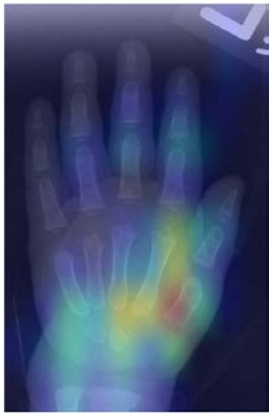	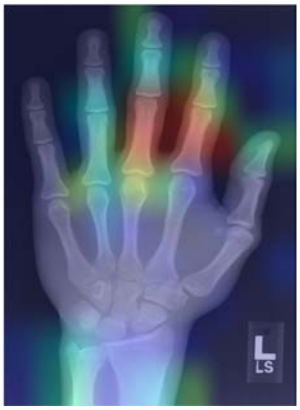
A2	Accurate primary race prediction - CT Chest	Black	White
		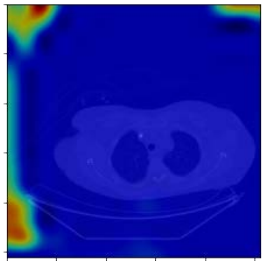	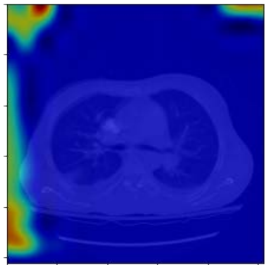
	Incorrectly classified race	Black incorrectly classified as White	White incorrectly classified as Black

	prediction - CT Chest	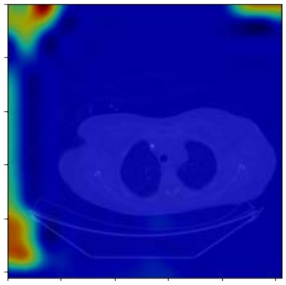	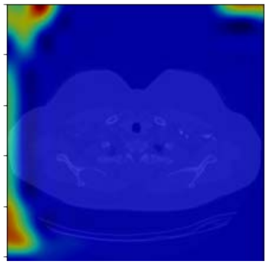
A2	Accurate race prediction - C Spine radiographs	Black	White
		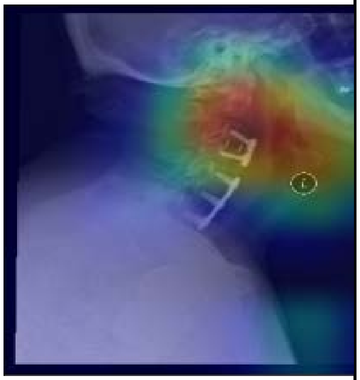 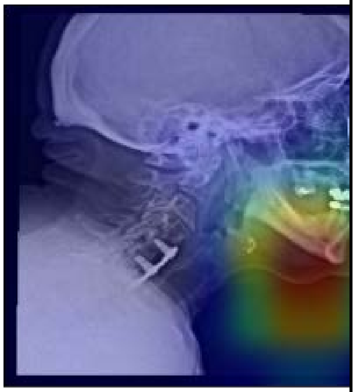	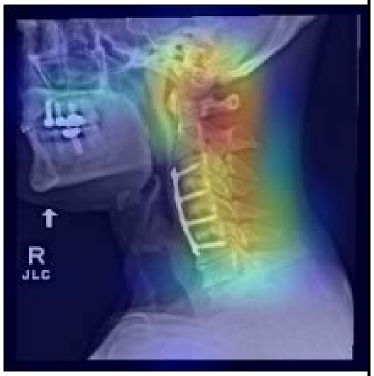 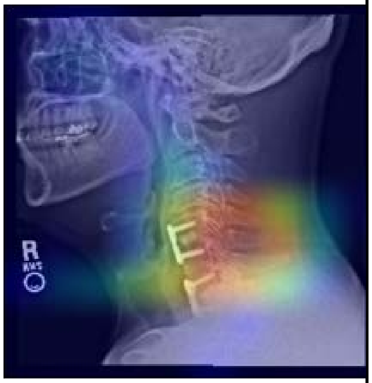
	Incorrectly classified race prediction - C Spine radiographs	Black incorrectly classified as White	White incorrectly classified as black
		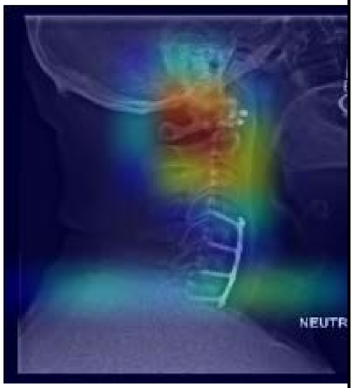	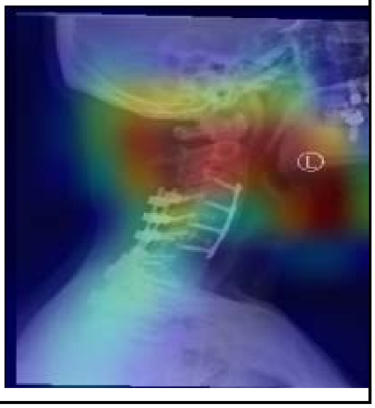

		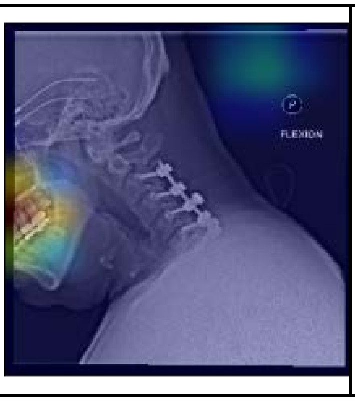	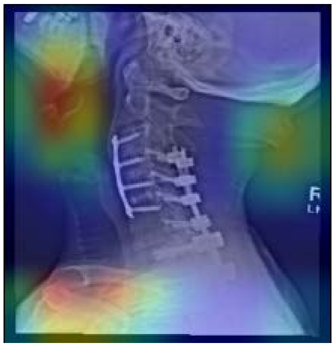

Figure S12 showing a sample CXR with the overall average pixel threshold intensities on the MIMIC-CXR dataset (Normal (0 - 255), Air (30 - 255), Fat (80 - 255), Soft Tissue (110 - 255) and Bone (180 - 255)

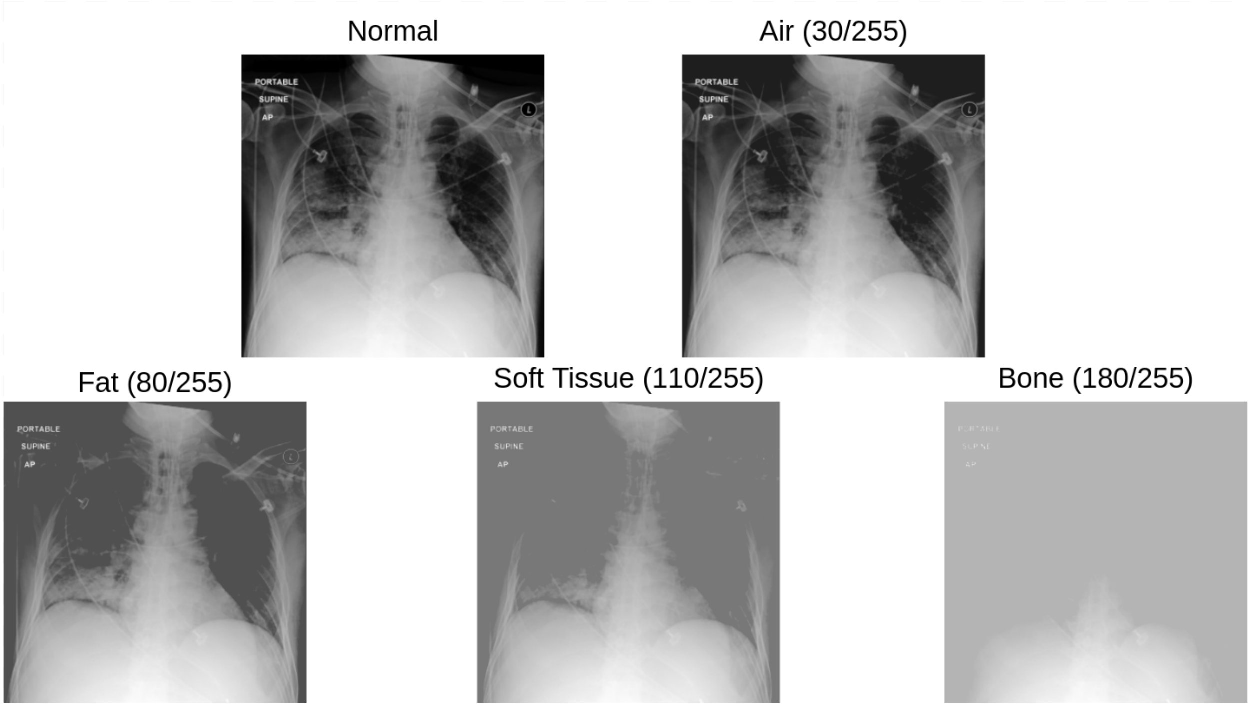

Figure S13 showing distribution of mammogram 2D Views by race across multiple hospital locations.

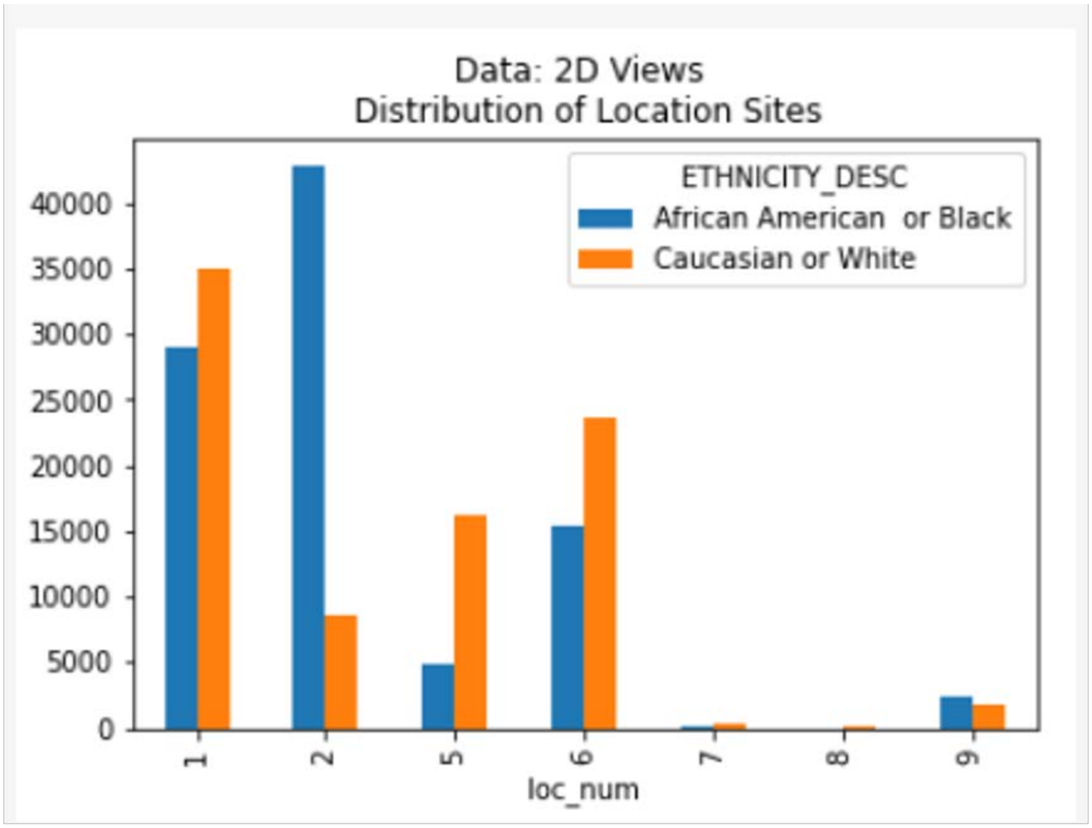
Data: 2D Views
Distribution of Location Sites
ETHNICITY_DESC
African American or Black
Caucasian or White
40000
35000
30000
25000
20000
15000
10000
5000
0
1
2
5
6
7
8
9
loc_num

Figure S14 showing distribution of mammogram 2D Views by race across multiple equipment manufacturers

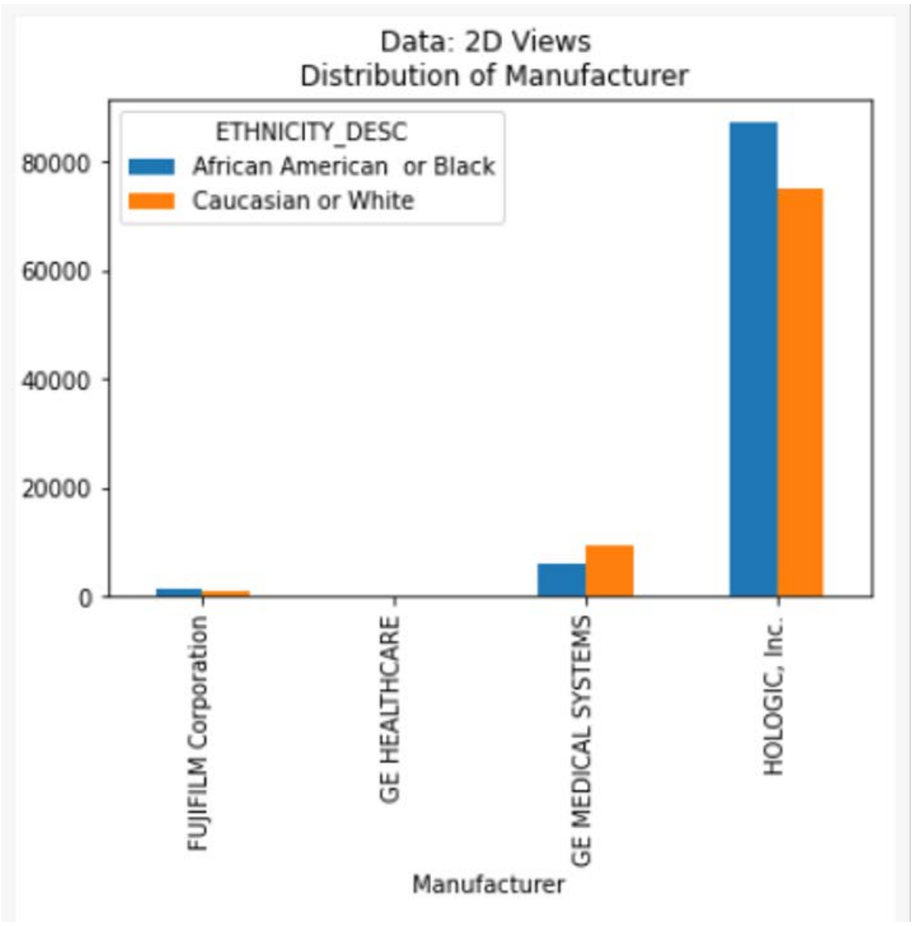

Figure S15 showing a sample CXR from the CheXpert dataset that has been digitally acquired with a smartphone camera.

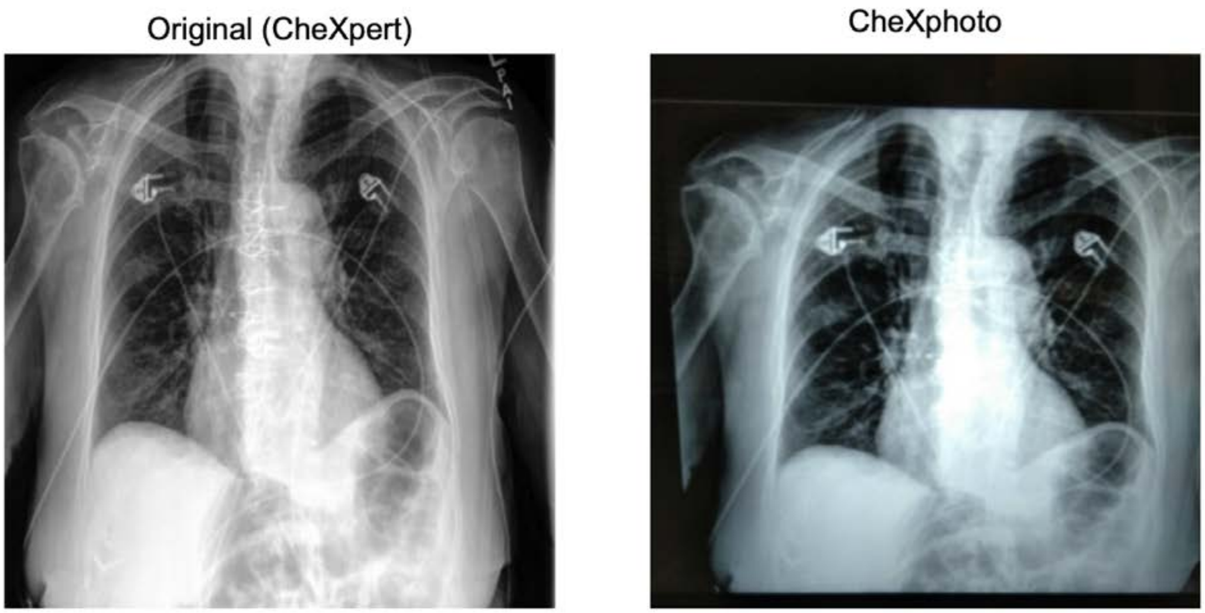

Figure S16 showing AUC curves for binary race prediction using combination of age, sex, disease and body habitus using logistic regression, random forest and XGBoost

GLM: Confidence interval for the score: [0.640 - 0.669]

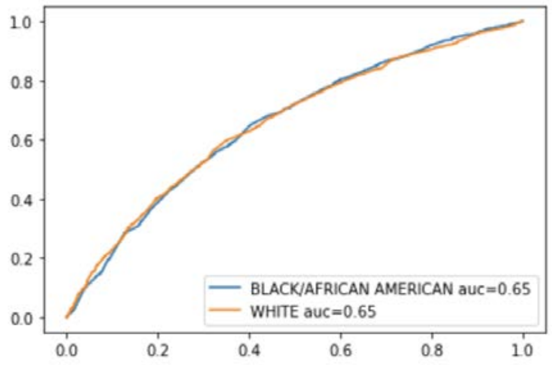

Random Forest: Confidence interval for the score: [0.647 - 0.676]

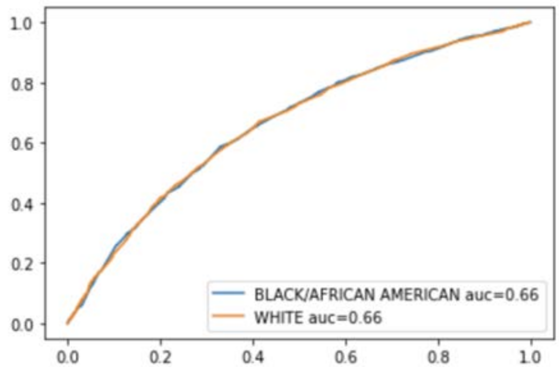

XGBoost:Confidence interval for the score: [0.663 - 0.689]

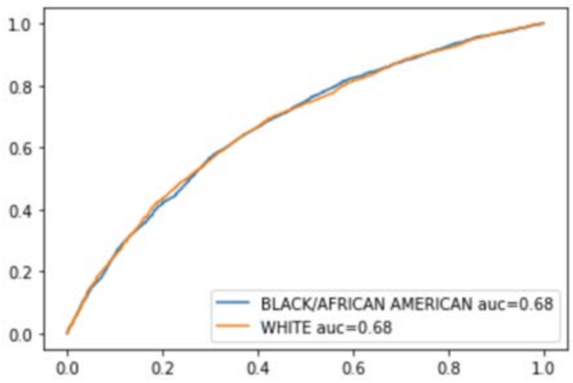

Table S1: Summary of model training details.

	A) Race detection in imaging								
	**MODELS**	**Pretrain**	**Input size**	**Optim.**	**Loss fn**	**LR**	**Batch size**	**Epochs**	**D/ Out**
A1	CXR models<br>● ResNet34<br>● Densenet121<br>● EfficientNetB0	 IN<br>IN<br>IN	 320x320<br>224x224<br>224x224	 Adam<br>Adam<br>Adam	 CCE<br>CCE<br>CCE	 1e-3<br>1e-3<br>1e-3	 256<br>128<br>256	 12-16<br>10<br>20	 No<br>0.5<br>0.4
A2	CT chest	IN	512x512	SGD w/ mom.	BCE	1e-4<br>1e-5	16	~...	
	Limb x-ray (IV) - DHA<br>● ResNet50	IN	320x320	Adam	CCE	1e-5	8	100	No
	Mammography<br>● EfficientNetB2	IN	256x256	Adam	BCE	1e-3	32	10 (ES)	No
	Cervical spine x-ray<br>● ResNet34	IN	320x320	Adam	CCE	1e-3	64	12-16 (ES)	No

	B) Experiments on clinical confounders	
	**MODELS**	**Parameters**
B1	BMI<br>● LR<br>● BMI stratified training and testing on MXR<br>● BMI subset analysis on EMX	 NA<br>CXR Densenet121 as above<br><br>NA
B2	Breast density<br>● LR<br>Breast density + Age<br>● LR	 NA<br><br>NA
B3	Disease distribution<br>● LR<br>● RF<br>● XGBoost<br><br>Image-based race detection for the "no finding" class	Disease distribution<br>● LR: L1 regularization, searching C ∈ [10^{-5}, 10^{1}], all other hyperparameters at default values from the scikit-learn library<br>● RF: 100 estimators, searching max depth ∈ {1, 2, ⋯, 6}, all other hyperparameters at default values from the scikit-learn library<br>● XGBoost: 100 estimators, searching max depth ∈ {1, 2, ⋯, 6}, all other hyperparameters at default values from the xgboost library<br><br>We trained and tested the Densenet121 model using the 35,307 and 18,362 images with "no finding" labels in the MXR dataset, respectively

B4	Bone density	CXR Densenet121 as above
B5	Impact of Age<br><br>Impact of patient sex	CXR Densenet121 as above<br><br>CXR Densenet121 as above
B6	Combination of age, sex, disease, and body habitus	Logistic regression model, random forest classifier, XGBoost model as above (B3)

C) Experiments to evaluate the mechanism of race detection		
	**Experiments**	**MODELS**
C1	Frequency domain imaging features	CXR Densenet121 as above
C2	Image resolution and quality	• Image resolution - CXR Resnet34 as above<br>○ We resized the MIMIC-CXR (MXR) images into 320x320, 240x240, 160x160, 80x80, 60x60, 40x40, 32x32, 24x24, 16x16, 8x8, 4x4, 2x2, and 1 pixel resolution.<br>○ split the training, validation and testing groups by patient ID by 80%, 10%, 10% respectively.<br>• Noisy and image perturbations - CXR Densenet121 as above
C3	Anatomical localisation<br><br>• Lung segmentation experiments<br>○ Seg model<br>○ Classification model<br><br>• Saliency maps<br><br>• Occlusion experiments<br><br>• Slice-wise results	 <br>The numbers of segmented CXRs used for testing are 148, 382, and 200 for White, Black, and Asian patients respectively<br>TernausNet.<br>As above...<br><br>Grad cam (keras)<br><br>CXR Densenet121 as above<br><br>N/A
C4	Patch-based training	CXR Densenet121 as above
C5	Image acquisition differences	EM-Mammo EfficientNetB2 as above

Table S2: Data distribution across four BMI groups (Underweight: <18.5, Normal: 18.5 to < 25, Overweight: 25 to < 30 and Obese > 30) in the MXR dataset showing the train/test split for experiment B1.

	Obese (BMI > 30)		Overweight (BMI 25 to < 30)		Normal (BMI 18.5 to < 25)		Underweight (BMI <18.5)	
	Train	Test	Train	Test	Train	Test	Train	Test
White	5262	2895	5591	2498	5392	2655	636	251
	25.8%	28.8%	27.5%	24.9%	26.5%	26.5%	3.1%	2.5%
Black	974	562	815	302	746	381	134	128
	4.8%	5.6%	4%	3%	3.7%	3.8%	0.7%	1.3%
Asian	57	33	444	168	256	156	50	8
	0.3%	0.3%	2.2%	1.7%	1.3%	1.6%	0.2%	0.08%

Note: Chi-square test implies that the two factors (BMI and Race) are not independent ($p < 0.05$).

Table S3: AUC values and confidence intervals of race detection in four BMI groups after stratified training and testing of a Densenet121 model on the MXR dataset using data splits in Table S2.

	Obese (BMI > 30)	Overweight (BMI 25 to < 30)	Normal (BMI 18.5 to < 25)	Underweight (BMI <18.5)
White	0.923 (0.909-0.936)	0.931 (0.918-0.944)	0.903 (0.884-0.922)	0.956 (0.935-0.977)
Black	0.930 (0.917-0.942)	0.964 (0.954-0.974)	0.885 (0.858-0.912)	0.966 (0.948-0.984)
Asian	0.914 (0.859-0.968)	0.918 (0.893-0.942)	0.940 (0.923-0.957)	0.976 (0.943-1.00)

Table S4: Results of subset analysis of a trained race detection model on the Emory CXR (EMX) dataset and subset analysis of race AUCs across four different BMI categories.

	Obese (BMI > 30)	Overweight (BMI 25 to < 30)	Normal (BMI 18.5 to < 25)	Underweight (BMI <18.5)
White	0.99	0.98	0.97	0.97
Black	0.99	0.99	0.98	0.98
Asian	0.94	0.96	0.93	0.92

Table S5: Data distribution across four breast density groups in the EM-Mammo dataset showing the train/test split for experiment B2. The dataset was split into training (16,296 patients), validation (5,432 patients), and testing (5,432 patients). Four groups of breast density were available in the dataset - (1 - fatty, 2 - scattered fibroglandular density, 3 - heterogeneously dense and 4 - extremely dense breasts). Most patients have scattered and heterogeneous breast density. There was no difference across the racial subgroups.

	1 (Fatty)		2 (Scattered)		3 (Heterogeneous)		4 (Dense)	
	Train	Test	Train	Test	Train	Test	Train	Test
White	2,594 (3%)	888 (1.0%)	13,007 (15.1%)	4,345 (5.0%)	14,758 (17.1%)	4,798 (5.5%)	1,800 (2.1%)	615 (0.7%)
Black	4,341 (5%)	1,405 (1.6%)	15,000 (17.3%)	5,003 (5.7%)	11,936 (13.8%)	4,061 (4.7%)	1,298 (1.5%)	463 (0.5%)

Note: Chi-square test implies that the two factors (Density and Race) are not independent ($p < 0.05$).

Table S6: Slice and study AUC values of race prediction across four breast density classes, and the overall dataset not split by breast density. There is no difference between the AUC values at various densities, and also between slice versus study AUC values.

Tissue Density	ROC AUC (Slice)	ROC AUC (Study)
1 (Fatty)	0.79 (0.765 - 0.806)	0.81 (0.776 - 0.842)
2 (Scattered)	0.78 (0.773 - 0.793)	0.82 (0.801 - 0.834)
3 (Heterogeneous)	0.77 (0.754 - 0.775)	0.80 (0.781 - 0.815)
4 (Dense)	0.72 (0.688 - 0.755)	0.74 (0.681 - 0.791)
Overall	0.78 (0.773 - 0.786)	0.81 (0.794 - 0.818)

Table S7: Race detection performance (as AUROC for white patients) using bandpass filtering on MXR for various values of d_1 and d_2. We observe that race information is present on all examples of transformed images even when barely perceptible to the human as a CXR.

**d_1 | d_2**	**25**	**50**	**75**	**100**	**125**	**150**
**10**	0.86	0.90	0.91	0.91	0.91	0.91
**25**		0.86	0.89	0.90	0.90	0.91
**50**			0.87	0.89	0.89	0.89
**75**				0.85	0.86	0.87
**100**					0.84	0.84

**125**						0.75

Table S8: Race detection performance (as AUROC for white patients) using notch filtering on MXR for various values of d_1 and d_2. We observe that race information is present on all examples of transformed images even when barely perceptible to the human as a CXR.

**d_1 | d_2**	**25**	**50**	**75**	**100**	**125**	**150**
**10**	0.90	0.89	0.87	0.85	0.82	0.82
**25**		0.90	0.90	0.89	0.89	0.89
**50**			0.91	0.91	0.91	0.90
**75**				0.91	0.91	0.91
**100**					0.91	0.91
**125**						0.91

Table S9: Comparative predictions using multiple architectures for primary race prediction on the MXR, EMX and CXP datasets. High AUCs are observed for Whites, Blacks and Asians across the three model architectures - Resnet34, Densenet121 and EfficientNetB0.

**Experiments**	**AUC of Race Classification**		
	**Asian**	**Black**	**White**
MXR Densenet121	0.944 (0.938-0.950)	0.940 (0.937-0.942)	0.933 (0.930-0.936)
CXP Resnet34	0.981 (0.979 - 0.983)	0.980 (0.977 - 0.983)	0.980 (0.978 - 0.981)
EMX Densenet121	0.911 (0.907-0.916)	0.965 (0.962-0.968)	0.948 (0.944-0.952)
EMX EfficientNet-B0	0.95 (0.938 - 0.957)	0.99 (0.986 - 0.99)	0.98 (0.979 - 0.984)
EMX Resnet34	0.969 (0.961 - 0.976)	0.992 (0.991 - 0.994)	0.988 (0.986 - 0.989)

Table S10: Data distribution across five age groups in the MXR dataset including the train/test split for experiment B5.

Age (yrs)	0-20		20-40		40-60		60-80		80+	
	Train	Test	Train	Test	Train	Test	Train	Test	Train	Test
White	269	173	7,085	4,082	25,312	12,887	39,938	20,439	17,229	8,181
	0.2%	0.3%	6.1%	7%	21.7%	22%	34.3%	34.9%	14.8%	14%
Black	100	43	3,122	1,796	8,170	3,926	8,513	3,794	2,151	1,073
	0.09%	0.07%	2.7%	3.1%	7%	6.7%	7.3%	6.5%	1.8%	1.8%
Asian	20	20	491	279	1,176	657	1,986	947	843	318
	0.02%	0.03%	0.4%	0.5%	1%	1.1%	1.7%	1.6%	0.7%	0.5%

Note: Chi-square test implies that the two factors (Age and Race) are not independent ($p < 0.05$).

Table S11: AUC values and confidence intervals of race detection in each age group after training a Densenet121 model on the MXR dataset. The low prediction value on the 0-20 age group for the Asian class is likely due to the small dataset size which is <1%.

	0-20	20-40	40-60	60-80	80+
White	0.913 (0.866-0.961)	0.900 (0.890-0.909)	0.931 (0.926-0.936)	0.945 (0.941-0.948)	0.918 (0.908-0.928)
Black	0.946 (0.904-0.987)	0.907 (0.897-0.917)	0.942 (0.931-0.952)	0.950 (0.946-0.954)	0.928 (0.918-0.938)
Asian	0.843 (0.746-0.941)	0.915 (0.890-0.940)	0.945 (0.941-0.948)	0.959 (0.952-0.966)	0.931 (0.911-0.950)

Table S12: Data distribution for male and female groups in the MIMIC-CXR (MXR) dataset including the train/test split for experiment B5.

	Male		Female	
	Train	Test	Train	Test
White	50,765	25,378	39,068	20,384
	43.6%	43.3%	33.6%	34.8%
Black	9,244	4,177	12,832	6,455
	7.9%	7.1%	11%	11%
Asian	2,580	1,149	1,936	1,072
	2.2%	2%	1.7%	1.8%

Note: Chi-square test implies that the two factors (Sex and Race) are not independent ($p < 0.05$).

Table S13: AUC values and confidence intervals of race detection for males and females after training a Densenet121 model on the MIMIC-CXR (MXR) dataset described in Table S12 .

	Asian	Black	White
MXR Densenet121-Original	0.944 (0.938-0.950)	0.940 (0.937-0.942)	0.933 (0.930-0.936)
MXR Densenet121-Male	0.941 (0.933-0.949)	0.921 (0.916-0.926)	0.914 (0.909-0.919)
MXR Densenet121-Female	0.951 (0.942-0.959)	0.953 (0.950-0.956)	0.948 (0.945-0.959)

Table S14: AUC values and confidence intervals of race detection at various image resolutions from 1 pixel resolution to 320x320.

Race	Resolution												
	1	2	4	8	16	24	32	40	60	80	160	240	320
Asian	0.634 [0.622 - 0.645]	0.573 [0.56 - 0.586]	0.678 [0.668 - 0.689]	0.707 [0.696 - 0.719]	0.744 [0.734 - 0.754]	0.76 [0.751 - 0.77]	0.815 [0.806 - 0.823]	0.811 [0.802 - 0.819]	0.888 [0.881 - 0.895]	0.919 [0.913 - 0.925]	0.962 [0.958 - 0.966]	0.972 [0.969 - 0.975]	0.986 [0.984 - 0.988]
Black	0.541 [0.536 - 0.548]	0.55 [0.544 - 0.556]	0.627 [0.621 - 0.633]	0.686 [0.681 - 0.692]	0.735 [0.729 - 0.74]	0.765 [0.76 - 0.77]	0.827 [0.823 - 0.832]	0.838 [0.834 - 0.842]	0.900 [0.897 - 0.903]	0.928 [0.925 - 0.931]	0.965 [0.963 - 0.967]	0.970 [0.968 - 0.972]	0.982 [0.981 - 0.983]
White	0.533 [0.527 - 0.538]	0.557 [0.552 - 0.563]	0.623 [0.618 - 0.628]	0.681 [0.675 - 0.686]	0.726 [0.721 - 0.731]	0.757 [0.752 - 0.762]	0.819 [0.815 - 0.823]	0.828 [0.824 - 0.832]	0.894 [0.891 - 0.897]	0.921 [0.918 - 0.924]	0.962 [0.96 - 0.964]	0.967 [0.965 - 0.969]	0.986 [0.984 - 0.988]

Table S15: AUCs and confidence intervals for race detection using noisy and blurred CXR images on the MXR dataset. The AUCs of the noisy and blurred values show a drop in performance, although the AUCs are > 0.50 (random chance) implying that some race information is still present in these images.

	Asian	Black	White
MXR Densenet121-Original	0.944 (0.938-0.950)	0.940 (0.937-0.942)	0.933 (0.930-0.936)
MXR Densenet121-Noisy	0.637 (0.625-0.650)	0.722 (0.716-0.728)	0.697 (0.691-0.702)
MXR Densenet121-Blurred	0.594 (0.581-0.607)	0.638 (0.631-0.644)	0.615 (0.609-0.621)

Table S16: AUCs and confidence intervals for race detection after masking the regions of interest indicated by the saliency maps. The AUCs decreased when the regions in the CXRs with the highest attention by the model were blocked out, but still maintained more than random chance of race detection.

	Asian	Black	White
MXR Densenet121-Original	0.944 (0.938-0.950)	0.940 (0.937-0.942)	0.933 (0.930-0.936)
MXR Densenet121-Masked	0.670 (0.665-0.676)	0.674 (0.668-0.680)	0.834 (0.823-0.841)

Table S17: Comparative AUC values and confidence intervals for the entire non segmented CXR, non lung and lung segmentations. Lung segmentations have the least AUC values while the original images have the highest AUCs. Race information is likely a combination of information from all portions of the image.

	Asian	Black	White
MXR Densenet121-Original	0.944 (0.938-0.950)	0.940 (0.937-0.942)	0.933 (0.930-0.936)
MXR Densenet121-Non lung	0.922 (0.896-0.947)	0.915 (0.892-0.939)	0.896 (0.868-0.924)
MXR Densenet121-Lung	0.734 (0.690-0.777)	0.724 (0.683-0.765)	0.731 (0.682-0.780)

Table S18: AUROC performance of classifiers trained on 14 binary disease labels to predict race in MXR and CXP. Classifiers used are XGBoost (XGB), L1-regularized logistic regression (LR) and random forest (RF).

	**MXR**			**CXP**		
	**XGB**	**LR**	**RF**	**XGB**	**LR**	**RF**
**White**	57.1%	56.9%	56.9%	52.1%	51.9%	51.9%
**Black**	60.8%	60.6%	60.5%	56.9%	56.6%	56.8%
**Asian**	56.1%	54.8%	56.8%	54.3%	54.2%	54.2%

Table S19: Performance of deep learning models on race prediction for MXR (AUROC for White vs. others) when a particular patch is removed from training by setting pixel intensities to zero. Quadrants shown correspond to the geometric location of the patch (e.g. quadrant (1,1) corresponds to the top left portion of the image).

**Quadrant**	**1**	**2**	**3**
**1**	0.91	0.90	0.91
**2**	0.91	0.91	0.91
**3**	0.91	0.91	0.91

Table S20: Performance of deep learning models on race prediction for MXR (AUROC for White vs. others) when only one of the nine patches is used for modeling.

**Quadrant**	**1**	**2**	**3**
**1**	0.87	0.88	0.87
**2**	0.81	0.82	0.81
**3**	0.75	0.60	0.75

Table S21: Performance of deep learning models on race prediction on the EM-Mammo (AUROC for Black versus White) across single locations and single equipment type.

	AUC-ROC
Original Mammo Model	0.81
Location 1	0.87
Location 2	0.92
Location 3	0.91
GE medical equipment	0.90

Hologic equipment	0.91

Table S22: Performance of deep learning models on race prediction on the Emory CXR across single equipment type and multiple equipment and locations.

Experiments	Asian	Black	White
Original EMX Densenet 121 model	0.91 (0.907-0.916)	0.97 (0.962-0.968)	0.95 (0.944-0.952)
Single hospital, single equipment model (Model A)	0.914 (0.880 - 0.941)	0.981 (0.977 - 0.986)	0.976 (0.971 - 0.981)
Testing of Model A on images from multiple equipment and multiple hospitals	0.869 (0.852 - 0.886)	0.972 (0.969 - 0.974)	0.962 (0.958 - 0.965)

Table S23: Performance of deep learning models on race prediction on the CheXphoto versus CheXpert Dataset

Experiments	Asian	Black	White
CheXpert dataset	0.90 (0.858 - 0.933)	0.94 (0.911 - 0.956)	0.89 (0.865 - 0.917)
CheXphoto dataset	0.894 (0.857 - 0.928)	0.787 (0.734 - 0.836)	0.857 (0.825 - 0.890)

References

1. Johnson AEW, Pollard TJ, Berkowitz SJ, Greenbaum NR, Lungren MP, Deng C-Y, et al. MIMIC-CXR, a de-identified publicly available database of chest radiographs with free-text reports. Sci Data. 2019 Dec 12;6(1):317.

2. Irvin J, Rajpurkar P, Ko M, Yu Y, Ciurea-Ilcus S, Chute C, et al. Chexpert: A large chest radiograph dataset with uncertainty labels and expert comparison. In: Proceedings of the AAAI Conference on Artificial Intelligence. 2019. p. 590–7.

3. Gertych A, Zhang A, Sayre J, Pospiech-Kurkowska S, Huang HK. Bone age assessment of children using a digital hand atlas. Comput Med Imaging Graph. 2007 Jun;31(4-5):322–31.

4. National Lung Screening Trial Research Team, Aberle DR, Berg CD, Black WC, Church TR, Fagerstrom RM, et al. The National Lung Screening Trial: overview and study design. Radiology. 2011 Jan;258(1):243–53.

5. Colak E, Kitamura FC, Hobbs SB, Others. The RSNA pulmonary embolism CT (RSPECT) dataset. Radiol Artif Intell. 2021;

6. He K, Zhang X, Ren S, Sun J. Deep residual learning for image recognition. In: Proceedings of the IEEE conference on computer vision and pattern recognition. 2016. p. 770–8.

7. Huang G, Liu Z, Van Der Maaten L, Weinberger KQ. Densely connected convolutional networks. In: Proceedings of the IEEE conference on computer vision and pattern recognition. 2017. p. 4700–8.

8. Tan M, Le QV. EfficientNet: Rethinking Model Scaling for Convolutional Neural Networks [Internet]. arXiv [cs.LG]. 2019. Available from: http://arxiv.org/abs/1905.11946

9. Chollet F. Keras: The Python Deep Learning library [Internet]. Astrophysics Source Code Library. 2018. p. ascl:1806.022. Available from: https://ui.adsabs.harvard.edu/abs/2018ascl.soft06022C

10. Carter JV, Pan J, Rai SN, Galandiuk S. ROC-ing along: Evaluation and interpretation of receiver operating characteristic curves. Surgery. 2016 Jun;159(6):1638–45.

11. Collins GS, Reitsma JB, Altman DG, Moons KGM. Transparent Reporting of a multivariable prediction model for Individual Prognosis Or Diagnosis (TRIPOD): the TRIPOD Statement. Br J Surg. 2015 Feb;102(3):148–58.

12. Moons KGM, Altman DG, Reitsma JB, Ioannidis JPA, Macaskill P, Steyerberg EW, et al. Transparent Reporting of a multivariable prediction model for Individual Prognosis or Diagnosis (TRIPOD): explanation and elaboration. Ann Intern Med. 2015 Jan 6;162(1):W1–73.

13. Cumming G, Calin-Jageman R. Introduction to the new statistics: Estimation, open science, and beyond. Routledge; 2016.

14. Nelder JA, Wedderburn RWM. Generalized Linear Models. J R Stat Soc Ser A. 1972;135(3):370.

15. Tolles J, Meurer WJ. Logistic Regression: Relating Patient Characteristics to Outcomes. JAMA. 2016 Aug 2;316(5):533–4.

16. Chen T, Guestrin C. XGBoost: A Scalable Tree Boosting System. In: Proceedings of the 22nd ACM SIGKDD International Conference on Knowledge Discovery and Data Mining. New York, NY, USA: Association for Computing Machinery; 2016. p. 785–94. (KDD ’16).

17. Stern EJ, Frank MS, Godwin JD. Chest computed tomography display preferences. Survey of thoracic radiologists. Invest Radiol. 1995 Sep;30(9):517–21.

18. Geirhos R, Rubisch P, Michaelis C, Bethge M, Wichmann FA, Brendel W. ImageNet-trained CNNs are biased towards texture; increasing shape bias improves accuracy and robustness [Internet]. arXiv [cs.CV]. 2018. Available from: http://arxiv.org/abs/1811.12231

19. Hermann KL, Chen T, Kornblith S. The Origins and Prevalence of Texture Bias in Convolutional Neural Networks [Internet]. arXiv [cs.CV]. 2019. Available from: http://arxiv.org/abs/1911.09071

20. Ilyas A, Santurkar S, Tsipras D, Engstrom L, Tran B, Madry A. Adversarial Examples Are Not Bugs, They Are Features [Internet]. arXiv [stat.ML]. 2019. Available from: http://arxiv.org/abs/1905.02175

21. Wang H, Wu X, Huang Z, Xing EP. High-frequency component helps explain the generalization of convolutional neural networks. In: Proceedings of the IEEE/CVF Conference on Computer Vision and Pattern Recognition. openaccess.thecvf.com; 2020. p. 8684–94.

22. Selvaraju RR, Cogswell M, Das A, Vedantam R, Parikh D, Batra D. Grad-cam: Visual explanations from deep networks via gradient-based localization. In: Proceedings of the IEEE international conference on computer vision. 2017. p. 618–26.

23. Iglovikov V, Shvets A. TernausNet: U-Net with VGG11 Encoder Pre-Trained on ImageNet for Image Segmentation [Internet]. arXiv [cs.CV]. 2018. Available from: http://arxiv.org/abs/1801.05746

24. Phillips NA, Rajpurkar P, Sabini M, Krishnan R, Zhou S, Pareek A, et al. CheXphoto: 10,000+ Photos and Transformations of Chest X-rays for Benchmarking Deep Learning Robustness. In: Alsentzer E, McDermott MBA, Falck F, Sarkar SK, Roy S, Hyland SL, editors. Proceedings of the Machine Learning for Health NeurIPS Workshop. PMLR; 2020. p. 318–27. (Proceedings of Machine Learning Research; vol. 136).
